# Distribution dynamics and urbanization-related factors of Hantaan and Seoul virus infections in China between 2001 and 2020: A machine learning modelling analysis

**DOI:** 10.1016/j.heliyon.2024.e39852

**Published:** 2024-10-29

**Authors:** Yao Tian, Tao Wang, Jin-Jin Chen, Qiang Xu, Guo-Lin Wang, Bao-Gui Jiang, Li-Ping Wang, Chen-Long Lv, Tao Jiang, Li-Qun Fang

**Affiliations:** a State Key Laboratory of Pathogen and Biosecurity, Academy of Military Medical Sciences, Beijing, 100071, China; b The 949th Hospital of Chinese PLA, Altay, Xinjiang, 836300, China; c Chinese Center for Disease Control and Prevention, Beijing, 102200, China

**Keywords:** HFRS, XGBoost, SHAP, Urbanization, Risk factor

## Abstract

**Objectives:**

The epidemical and clinical features of distinct hantavirus infections exhibit heterogeneity. However, the evolving epidemics and distinct determines of the two hantavirus infections remain uncertain.

**Methods:**

Data on hemorrhagic fever with renal syndrome (HFRS) cases and genotyping were collected from multiple sources to explore the distribution dynamics of different endemic categories. Four modelling algorithms were used to examine the relationship between infected hantavirus genotypes in HFRS patients, as well as assess the impacts of urbanization-related factors on HFRS incidence.

**Results:**

The number of cities dominated by Hantaan (HTNV) and Seoul (SEOV) viruses was projected to decrease between two phases, while the mixed endemic cities increased. Patients with SEOV infection predominantly presented gastrointestinal symptoms. The modeling analysis revealed that built-up land and real GDP demonstrated the highest contribution to HTNV and SEOV infections, respectively. The impact of nightlight index and park green land was more pronounced in HTNV-dominant cities, while cropland, impervious surface, and floor space of commercialized buildings sold contributed more to HFRS incidence in SEOV-dominant cities.

**Conclusions:**

Our findings fill a gap for the three endemic categories of HFRS, which may guide the development of targeted prevention and control measures under the conditions of urbanization development.

## Highlights

• There were many differences between HTNV-dominant cities and SEOV-dominant cities in terms of their distribution range, clinical manifestation and urbanization-related factors.
• The conversion between endemic categories from 2001 to 2010 to 2011–2020 mainly took place in inland areas.
• Patients with SEOV infection predominantly presented gastrointestinal symptoms.
• Five urbanization-related factors were found to contribute to both the two genotype infections with distinct effects and lags, i.e., built-up land, population density, green land, real GDP and impervious surface.

## 1 Introduction

Hemorrhagic fever with renal syndrome (HFRS) is a zoonotic disease caused by hantaviruses, which are primarily transmitted to humans through inhalation of aerosols contaminated with virus shed in excreta, saliva, and urine from infected rodents. Clinical symptoms in infected patients are primarily characterized by fever, bleeding, hypotension, headache, back pain, abdominal pain, and acute renal insufficiency. HFRS is currently widespread in regions including China, the Korean Peninsula, Japan, and the Far East of Russia, among which China accounts for about 70–90 % of the total number of HFRS cases reported globally [1], primarily attributed to Hantaan virus (HTNV) and Seoul virus (SEOV), two strains of hantaviruses: HTNV carried by wild rodents such as *Apodemus agrarius* and *Apodemus peninsulae*, and Seoul virus (SEOV) carried by commensal rodents like *Rattus norvegicus* and *Mus musculus*. The case fatality rate of this disease varies between 0.43 % and 15 %, partly dependent on the viral strains. Between 1950 and 2018, a staggering disease burden was observed as over 1.67 million cases were reported across more than 300 Chinese cities.

HFRS is considered as one of the highly monitored infectious diseases in China, exhibiting a seasonal peak during Autumn-Winter or Spring, or even dual peaks within a single year, which is associated with the epidemic of distinct hantavirus genotypes [2]. Currently, there are 41 state-level HFRS surveillance sites established nationwide covering the majority of high-endemic regions [3]. However, compared to the extensive endemic areas in China, there remains a relatively limited amount of surveillance data on viral genotyping. Consequently, our understanding of the types of epidemic focus, dynamic changes, and ecological determinants associated with the dominant hantavirus genotypes in most endemic regions of the country is still inadequate.

With rapid urbanization, the environmental changes and population movement, acknowledged as influential factors driving communicable disease transmission, have greatly affected the diversity of rodent species, populations, and behavioral patterns [4]. In recent years, Guangzhou, as a representative city experiencing rapid urbanization, has witnessed an increasing trend in the number of HFRS cases, attributed to changes in land use, population migration, and the formation of urban villages [5]. Not only that, in traditional HFRS endemic areas like Shandong Province, the endemic scopes of SEOV were expanding from western to eastern regions, as a result of the inappropriate universal deratization and environmental changes during urbanization [6].

The process of urbanization development has been demonstrated to fragment the rodent habitats and compel rodents to seek suitable living conditions in urban areas, thereby potentially increasing human-rodent contact opportunities [7,8]. It has been demonstrated in recent studies that the establishment of newly constructed parks and green spaces can further facilitate the proliferation of rodent populations and pose concealed risks for disease transmission [9,10]. Therefore, it is imperative to conduct a comprehensive study into the association between the urbanization and different hantavirus infections. Especially, it is crucial to undertake specific analysis and comparison while considering the distinct virus genotypes.

The present study aimed to investigate the spatiotemporal distribution, suitable ecological niches, and clinical manifestations of different hantavirus genotypes infections, by using a comprehensive database of hantavirus genotypes identified in humans and rodents. Machine learning models were employed to assess and compare the impact of urbanization-related factors on the epidemic dynamics of HFRS in HTNV-dominant, SEOV-dominant, and mixed endemic areas, to provide guidance for targeted surveillance and prevention strategies under the background of rapid urbanization development.

## 2 Methods

### 2.1 Data collection and management

A comprehensive database of hantavirus genotypes identified in humans and rodents was compiled by integrating data from diverse sources such as literature review, the GenBank database, regular annual surveillance for selected notifiable infectious diseases, and the national etiology surveillance program for febrile hemorrhagic syndrome. We conducted an extensive literature search across multiple databases, including PubMed, Web of Science, GenBank, China National Knowledge Infrastructure and WanFang database from January 1st, 2000 to December 31st, 2022 without any language restriction. Studies reporting hantavirus genotypes were retrieved from an extensive literature search using the following keywords to search titles, abstracts, and keywords: “Hantavirus” OR “Orthohantavirus” OR “Hantaan virus” OR “Seoul virus” OR “HTNV” OR “SEOV”, combined with the term “China”. The titles and abstracts of all studies were independently screened for inclusion using Endnote (YT, JJC). The full text of all eligible studies was retrieved and assessed for eligibility by the same authors. Disagreements were resolved through discussions with a third author (CLL). The inclusion criterion for the literature screening stage consisted of field studies that reported the presence of HTNV or SEOV, providing explicit information on the study period, location (at least at the city level), and testing methods. The following criteria were applied for study exclusion: (I) lack of relevance in detecting hantavirus; (II) absence of city-level location information for detection; (III) falling outside the research period from 2001 to 2020; (IV) involvement in laboratory infection or infection experiments; or (V) focus on drug or vaccine development, systematic reviews, meta-analyses, opinions and perspectives, or conference posters and presentations. The confirmatory tests for hantavirus genotypes include the indirect immunoinfluscent assay (IFA), neutralization assay, reverse transcription-polymerase chain reaction (RT-PCR), and next generation sequencing (NGS). The extracted information includes the study date, study location, host species (animal or human), hantavirus genotypes, laboratory testing methods, and hantavirus detection results.

In addition, regular annual surveillance for selected notifiable infectious diseases, and the national etiology surveillance program for febrile hemorrhagic syndrome were also included in our comprehensive database. The details of those and the process of constructing the database were described in the appendix pp 1–3. In cases where multiple species of hantavirus were identified, a separate record was generated for each hantavirus species within our database. To facilitate a thorough genotyping analysis at the city level, data from various sources were integrated into a unified database. Furthermore, we have included data on laboratory- and clinically-confirmed cases of HFRS at the city level from January 1st, 2001 to December 12th, 2020. These records were obtained through the China Information System for Disease Control and Prevention (CISDCP). The aim is to examine the spatiotemporal dynamics of HFRS occurrences and assess the impact of urbanization-related factors on HFRS incidence.

For modelling analysis of geographic distribution dynamics of HTNV and SEOV infections, a total of 40 ecological factors were collected at the city level, including 10 biological, 19 ecoclimatic and 11 environmental variables. Details on the spatial resolution, study duration and source of the included data were provided in the appendix (pp 3–5 and Supplementary Table 1). The inclusion of variables was based on their relevance to the ecology and transmission dynamics of hantaviruses (Supplementary Table 2).

To assess the impact of urbanization-related factors on HFRS incidence in areas endemic to HTNV-dominant and SEOV-dominant strains, we incorporated ten factors related to urbanization that have been recognized as representative of various aspects of urban development (Supplementary Tables 1 and 2). In addition, we also included three types of land cover (cropland, forest, and impervious surface per capita) in this analysis, as which constitute a significant proportion across all land cover categories and exhibit strong correlation with urbanization activities [11,12].

### 2.2 Modelling analysis for geographic distribution dynamics of HTNV and SEOV infections

Considering potential shift of hantavirus genotypes during the 20-year study period, we divided the study period into two phases: 2001–2010 and 2011–2020. A two-stage extreme gradient boosting (XGBoost) model was conducted to predict the potential endemic types (HTNV-dominant, SEOV-dominant, and mixed endemic cities) for cities without available genotyping information in each phase (appendix pp 5–6). In the two-stage XGBoost modelling analysis, we included 11 environmental, 19 bioclimatic, and 10 biological variables, as well as real gross domestic product (GDP) per capita and population density as explanatory variables (Supplementary Tables 1 and 2). To mitigate multicollinearity among variables and evaluate the performance of the XGBoost model, we applied variance inflation factor (VIF) and 10-fold cross-validation during the modelling process. In the first stage, a XGBoost model was constructed to project the probabilities of HTNV and SEOV occurrences which were included in the second stage analysis as two additional predictors. In the second stage, the projected proportion of HTNV occurrence was estimated for all cities that have reported hantavirus occurrence and those identified at risk of HTNV or SEOV occurrence during the initial stage modelling analysis. Subsequently, these cities were classified into HTNV-dominant, SEOV-dominant, and mixed endemic cities for the two study phases by the projected proportion of HTNV occurrence, respectively. In order to validate the model's performance, we selected all cities with over 10 cumulative detection records of hantavirus genotypes during 2011–2015 (or 2006–2010), and categorized each city as HTNV-dominant (proportion of HTNV≥80 %), or SEOV-dominant (proportion of HTNV≤20 %), or mixed endemic one (proportion of HTNV between 20 % and 80 %) based on the proportion of HTNV detection records, and then we used the model established in Phase I (or II) to project the proportions of HTNV for these cities, which allowed us to redefine their endemic types using the same criteria. Details of the model construction were provided in the appendix (pp 7–8).

### 2.3 Comparative analysis of clinical characteristics of patients infected with HTNV and SEOV

The clinical and demographic data of patients infected with HTNV and SEOV was extracted from 835 case reports. We conducted a comparative analysis of the clinical spectrum observed in patients infected with HTNV and SEOV. The *t*-test was applied to compare continuous variables, and Pearson's Chi-square test or Fisher's exact test was used for the comparison of categorical variables. Based on clinical characteristics and locations of hantavirus infection, infections with HTNV or SEOV were differentiated by constructing four models, including the XGBoost model, random forest (RF), gradient boosting machine (GBM), and generalized linear model (GLM) (Appendix pp 8–10). We incorporated 25 clinical symptoms and two clinical laboratory tests (white blood cell count and platelet count) indicators, along with the projected proportion of HTNV infection in the respective city where the case was located from the previous two-stage model, as explanatory variables while adjusting for age and sex. After evaluating multiple models, we identified the one with the best performance. The Shapley Additive Explanations (SHAP) method was used to evaluate the individual contribution of each independent variable (appendix p 6).

### 2.4 The analysis of the association between HFRS incidence and urbanization-related factors

The association between HFRS incidence and urbanization-related factors was examined by conducting four machine learning models widely used in epidemiological studies, including XGBoost, RF, GBM and GLM. The aforementioned models were separately applied to HTNV-dominant and SEOV-dominant endemic cities projected by the two-stage model during either 2001–2010 or 2011–2020. The annual HFRS incidence for each city was used as the response variable and 10 urbanization-related factors and three land cover variables were used as independent variables in these models, along with 19 ecoclimatic factors as covariates. Least absolute shrinkage and selection operator (LASSO) regression analysis was conducted to select the inclusion of urbanization-related variables and their associated lag terms using the R package "glmnet", while keeping the ecoclimatic covariates and autocorrelation term fixed in the model. The best model performance was subsequently achieved through model evaluation and comparison. Based on this, the pooled exposure-response curves were plotted. Details of the modelling analysis were given in appendix (pp 10–11).

## 3 Results

A total of 2545 unique publications were retrieved from the literature search. Among them, 2378 publications were excluded based on the exclusion criteria (Supplementary Fig. 1). The remaining 167 publications contributed to a total of 6543 records regarding the hantavirus genotypes (Supplementary Table 3 and Supplementary reference 2). Additionally, we collected an additional 2722 records from GenBank (50 records), regular annual surveillance for selected notifiable infectious diseases (1837 records), and the national etiology surveillance program for febrile hemorrhagic syndrome (835 records). These combined sources formed the unified database (9265 records) for our study.

### 3.1 Geographic distribution dynamics of HTNV and SEOV infections

**Fig. 1:**
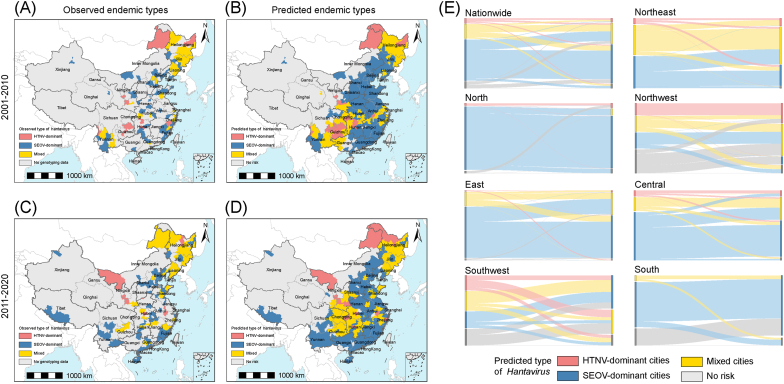
**Geographic distribution dynamics and conversion of three endemic categories for HFRS in the mainland of China, 2001**–**2020.** The observed and projected geographic distribution dynamics of the three endemic categories are presented by two phases: 2001–2010 (A, C) and 2011–2020 (B, D). The grey background indicates the areas with no genotyping data or no risk, and the other three colored backgrounds indicate the observed or projected areas of three endemic categories. The observed types of “HTNV-dominant” and “SEOV-dominant” indicate the areas where only HTNV and SEOV are reported, respectively, while type of “Mixed” indicates the areas where the two hantavirus genotypes are both reported. The predicted types of “HTNV-dominant”, “SEOV-dominant” and “Mixed” indicate the areas where the predictive proportions of HTNV are above 80 %, below 20 % or lying between, respectively. The regional conversion among the three endemic categories between the two phases is shown in (E).

Upon comparison, the external validation accuracies of the models in Phase I and II were 88.0 % (44/50) and 84.4 % (38/45), respectively, proving the good extrapolation performance of our models. from 2001 to 2010, a total of 91 cities with available hantavirus genotyping data were classified into three groups: HTNV-dominant (13 cities), SEOV-dominant (48 cities), and mixed endemic cities (30 cities). The number of genotyped cities increased to 109 during 2011–2020, including 9 HTNV-dominant, 60 SEOV-dominant, and 40 mixed endemic cities (Fig. 1A and C, Supplementary Fig. 2). Among these genotyped cities, the proportion of HTNV-dominant ones had decreased from 14.29 % to 8.26 % over the two study phases, while that of SEOV-dominant and mixed endemic cities increased from 52.75 % to 55.05 % and 32.97 %–36.70 %, respectively.

The estimated proportion of HTNV infection, as determined by the two-stage XGBoost model, was predominantly associated with the predicted probabilities of HTNV and SEOV occurrences provided by the initial stage model during both study phases (Supplementary Figs. 3 and 4). The SEOV-dominant cities exhibited the widest geographical distribution and had the largest population size at potential risk over the two decades, followed by mixed endemic cities and HTNV-dominant cities (Table 1). The population size potentially affected by HTNV decreased by 40 %, but the number of people at potential risk in mixed endemic cities increased by 52.16 %. During the first study phase, Southwest China had the highest number of HTNV-dominant cities (10 cities), covering an area of 168.65 thousand km^2^ and encompassing 38.38 million people, mainly distributed in Guizhou province. This was followed by Central China (4 cities), Northeast China (3 cities) and Northwest China (3 cities). On the other hand, East China had the majority of the SEOV-dominant cities (58 cities) with an area of 614.31 thousand km^2^ where nearly 300.22 million people reside, followed by Central China (32 cities), North China (28 cities) and South China (24 cities) (Fig. 1B). During the second study phase, there appeared to be a tend towards northward movement in the geographic distribution of HTNV infections, with no cities exhibiting dominance of HTNV in Southwest and South China. Additionally, there was a slight increase in the number of HTNV-dominant cities in Northwest (from 3 to 5) and Northeast China (from 3 to 4). The SEOV-dominant cities were predicted to be most widely distributed in East China (49 cities), while an increased risk of SEOV infections were observed in South and Southwest China. Relatively, the mixed endemic cities were predicted to be geographically concentrated in Northeast (20 cities), Southwest (16 cities) and Central (15 cities) China, while being scattered across East China (25 cities) as well (Fig. 1D).

**Table 1:** The areas and population size projected by XGBoost model at potential risk of different endemic categories.

	HTNV-dominant cities			SEOV-dominant cities			Mixed endemic cities		
	Number of cities	Area (1000 km^2^, %)	Population size (million, %)	Number of cities	Area (1000 km^2^, %)	Population size (million, %)	Number of cities	Area (1000 km^2^, %)	Population size (million, %)
2001–2010									
Nationwide	22	737.88 (7.67)	77.37 (5.81)	174	2347.29 (24.41)	844.93 (63.39)	61	1184.32 (12.31)	264.36 (19.84)
Northeast	3	150.13 (16.42)	4.53 (4.13)	17	279.43 (30.56)	52.66 (48.09)	16	484.87 (53.02)	52.33 (47.78)
North	1	313.70 (18.74)	2.55 (1.55)	28	634.88 (37.93)	152.13 (92.30)	0	0 (0)	0 (0)
Northwest	3	38.54 (1.23)	13.41 (13.88)	4	75.68 (2.41)	15.68 (16.23)	4	75.04 (2.39)	10.60 (10.96)
East	1	8.03 (1.07)	1.40 (0.36)	58	614.31 (82.05)	300.22 (76.42)	16	119.98 (16.03)	88.25 (22.46)
Central	4	58.84 (11.10)	17.10 (7.88)	32	352.09 (66.39)	162.49 (74.89)	9	109.68 (20.68)	33.76 (15.56)
Southwest	10	168.65 (7.73)	38.38 (19.89)	11	192.82 (8.84)	47.60 (24.67)	13	317.47 (14.56)	70.60 (36.58)
South	0	0 (0)	0 (0)	24	198.08 (49.58)	114.15 (71.78)	3	77.29 (19.34)	8.83 (5.55)
2011–2020									
Nationwide	17	925.17 (9.62)	46.16 (3.27)	163	2410.58 (25.06)	846.27 (60.03)	87	1399.50 (14.55)	402.24 (28.53)
Northeast	4	224.30 (24.53)	3.39 (3.44)	11	187.10 (20.46)	31.67 (32.15)	20	491.77 (53.78)	61.81 (62.74)
North	2	568.09 (33.94)	2.51 (1.48)	23	512.69 (30.63)	126.26 (74.56)	3	47.01 (2.81)	28.06 (16.57)
Northwest	5	68.70 (2.19)	20.54 (19.84)	2	41.57 (1.33)	4.85 (4.68)	8	152.97 (4.88)	23.75 (22.94)
East	2	20.08 (2.68)	9.12 (2.15)	49	521.92 (69.71)	281.57 (66.49)	25	198.64 (26.53)	131.43 (31.04)
Central	4	44.00 (8.30)	10.61 (4.74)	24	249.86 (47.12)	139.76 (62.52)	15	215.47 (40.63)	66.33 (29.67)
Southwest	0	0 (0)	0 (0)	22	571.03 (26.18)	93.23 (45.44)	16	293.64 (13.46)	90.85 (44.29)
South	0	0 (0)	0 (0)	32	326.41 (81.70)	168.93 (90.72)	0	0 (0)	0 (0)

The percentages in parentheses indicate the proportions of areas and population sizes at risk to the total area and population of the country or corresponding geographical divisions, respectively. The population sizes at risk and their proportions during 2001–2010 and 2011–2020 were calculated based on the 6th China Population Census in 2010 and the 7th China Population Census in 2020, respectively (http://www.stats.gov.cn/sj/pcsj/).

By conducting regional statistics on the conversion between endemic categories from 2001 to 2010 to 2011–2020, we found that the conversion mainly took place in inland areas, exhibiting significant regional heterogeneity in terms of direction (Fig. 1E). In comparison to the conversion from mixed endemic to SEOV-dominant, a higher number of cities were observed transitioning from the latter to the former, while there was greater variability in conversions among HTNV-dominant cities. In North China, it was mainly characterized by the conversion from SEOV-dominant to mixed endemic, with similar conversion modes observed in Central and East China. Northeast and Northwest China were the two representative regions showing a conversion from mixed endemic to HTNV-dominant. The conversion in South China was correspondingly simple, with the replacement of the original SEOV-dominant cities by some newly added ones.

### 3.2 Clinical characteristics of patients infected with HTNV or SEOV

A total of 835 patients with confirmed HFRS genotypes were recorded, including 143 (17.13 %) patients infected with HTNV and 692 (82.87 %) patients infected with SEOV (Table 2). Among them, there were no significant differences observed in terms of sex and age composition between the patients infected with the two hantavirus genotypes. It is worth noting that patients with HTNV infections exhibited a higher likelihood of experiencing skin flushing (neck), while those infected with SEOV most frequently presented gastrointestinal symptoms such as nausea, vomiting, bellyache, diarrhea and jaundice. Compared to the patients infected with SEOV, HTNV infections exhibited a higher proportion of positive urine protein. No significant differences were found between the two hantavirus genotype infections in relation to other symptoms or case fatality rates.

**Table 2:** Basic characteristics of patients infected with different *Hantavirus* genotypes.

Variables	HTNV		SEOV		p values
	No. of cases	Proportion (%)	No. of cases	Proportion (%)	
Demographic characteristics					
Sex					0.071
Male	96	67.13	518	74.86	
Female	47	32.87	174	25.14	
Age, years					0.530
0‒17	6	4.20	27	3.90	
18‒39	49	34.27	193	27.89	
40‒59	69	48.25	351	50.72	
60‒69	16	11.19	96	13.87	
≥70	3	2.10	25	3.61	
Clinical characteristics					
Death (Yes)	1	0.70	5	0.72	1.000
Symptoms					
Fever (≥38.5 °C)	100	69.93	438	63.29	0.155
Headache	77	53.85	401	57.95	0.418
Low back pain	70	48.95	344	49.71	0.941
Orbital pain	36	25.17	168	24.28	0.904
Arthralgia	27	18.88	188	27.17	0.050
Pantalgia	33	23.08	261	37.72	0.001
Nausea	59	41.26	379	54.77	0.004
Vomiting	31	21.68	244	35.26	0.002
Bellyache	22	15.38	240	34.68	<0.001
Diarrhea	17	11.89	169	24.42	0.002
Constipation	3	2.10	24	3.47	0.603
Skin flushing (face)	48	33.57	282	40.75	0.132
Skin flushing (neck)	28	19.58	88	12.72	0.043
Skin flushing (chest)	18	12.59	65	9.39	0.313
Hemoptysis	1	0.70	7	1.01	1.000
Conjunctival congestion	51	35.66	245	35.40	1.000
Eyelid edema	22	15.38	151	21.82	0.106
Jaundice	1	0.70	83	11.99	<0.001
Lymphadenopathy	3	2.10	9	1.30	0.442
Hepatosplenomegaly	5	3.50	26	3.76	1.000
Oliguria/anuria/haematuria	32	22.38	165	23.84	0.789
Mucosal hemorrhage (oral/nasal cavity)	17	11.89	102	14.74	0.449
Cutaneous hemorrhage	27	18.88	141	20.38	0.771
Rash	1	0.70	12	1.73	0.708
Petechiae/ecchymosis	8	5.59	34	4.91	0.897
Clinical laboratory tests (qualitative)					
Urine protein test					0.030
Positive	83	95.40	337	87.31	
Negative	4	4.60	49	12.69	
Tourniquet test					0.076
Positive	7	63.64	9	31.03	
Negative	4	36.36	20	68.97	
Bleeding time					0.306
Decrease	4	7.14	26	8.41	
Normal	18	32.14	130	42.07	
Increase	34	60.72	153	49.52	
Clotting time					0.773
Decrease	4	5.80	26	7.26	
Normal	28	40.58	155	43.30	
Increase	37	53.62	177	49.44	
Clinical laboratory tests (quantitative)	Mean	95%CI	Mean	95%CI	
White blood cell count (10^9^/L)	12.08	(10.79, 13.37)	11.22	(9.63, 12.81)	0.410
Platelet count (10^9^/L)	107.04	(89.18, 124.90)	100.24	(90.09, 110.38)	0.514

The p values were calculated by Pearson's Chi-square test or Fisher's exact test for the categorical variables and by *t*-test for the continuous variables.

**Fig. 2:**
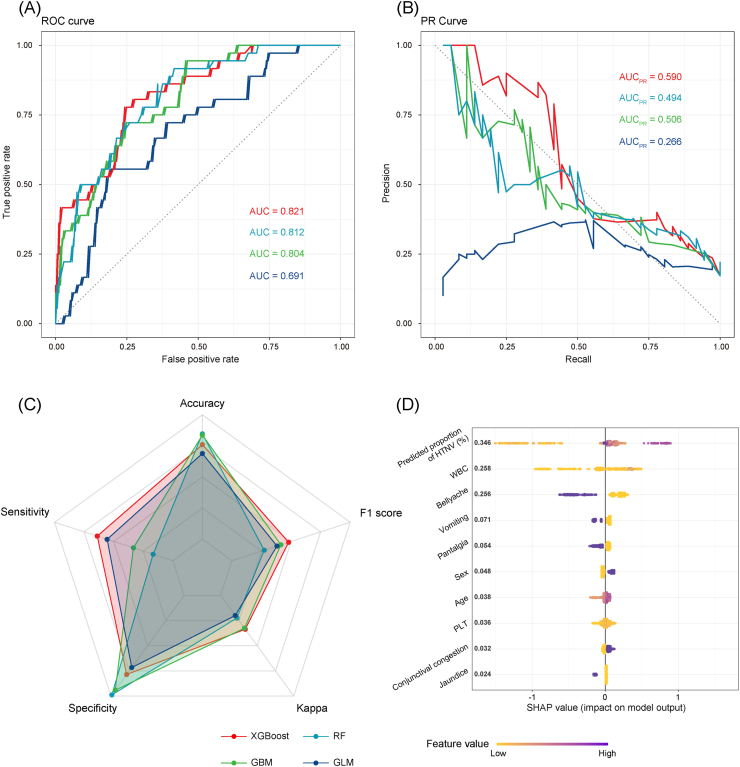
**Modelling analysis of infections with different hantavirus genotypes based on clinical characteristics.** (A) ROC curves of different models. (B) PR curves of different models. (C) Comparison of five evaluation indicators for different models. (D) Importance and effects of major variables indicated by SHAP values based on the optimal model (XGBoost). The variables are ranked in the importance according to their global SHAP values from top to bottom, with the y-axis indicating different variables and the x-axis indicating the SHAP values. Colors from yellow to purple indicate feature values from low to high. ROC: receiver operating characteristic. PR: precision-recall. SHAP: shapley additive explanations. XGBoost: extreme gradient boosting. AUC: area under curve. RF: random forest. GBM: gradient boosting machine. GLM: generalized linear model. WBC: white blood cell. PLT: platelet.

In the modelling analysis for differentiation of different hantavirus infections, XGBoost outperformed RF, GBM and GLM in terms of predictive performance, and demonstrated satisfactory performance in distinguishing HTNV or SEOV infections (Fig. 2A‒C). Based on the SHAP values, the projected proportion of HTNV infections in the corresponding city exhibited the most significant impact and demonstrated a positive association with the probability of patients being infected with HTNV, followed by white blood cell (WBC) count and bellyache (Fig. 2D). Male HFRS patients with lower WBC count, younger age, and residing in areas with a lower projected proportion of HTNV infections, accompanied by bellyache, vomiting, and pantalgia, were more likely to be SEOV infectors (Supplementary Fig. 5).

### 3.3 Heterogenous association of urbanization-related factors with HFRS incidence

**Fig. 3:**
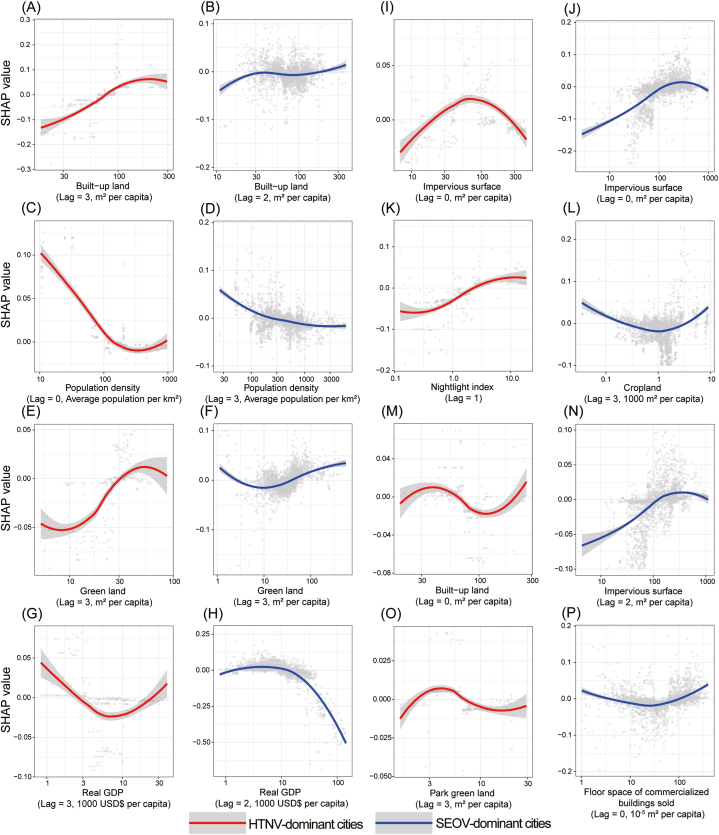
**Pooled exposure-response curves between HFRS incidence and urbanization-related factors in HTNV-dominant and SEOV-dominant cities during 2001**‒**2020 based on XGBoost model.** (A) Built-up land (lag = 3). (B) Built-up land (lag = 2). (C) Population density (lag = 0). (D) Population density (lag = 3). (E) Green land (lag = 3). (F) Green land (lag = 3). (G) Real GDP (lag = 3). (H) Real GDP (lag = 2). (I) Impervious surface (lag = 0). (J) Impervious surface (lag = 0). (K) Nightlight index (lag = 1). (L) Cropland (lag = 3). (M) Built−up land (lag = 0). (N) Impervious surface (lag = 2). (O) Park green land (lag = 3). (P) Floor space of commercialized buildings sold (lag = 0). Pooled exposure-response curves of HTNV**-**dominant and SEOV-dominant cities were plotted in red and blue lines, respectively, with their 95 % CI indicated by the shaded areas. The x-axis (plotted in log scale) indicates the observed values of the urbanization-related factors, while the y-axis indicates the SHAP value, with the scattered points indicating the distribution of fitting results for each city every year. GDP: gross domestic product. CI: confidence interval.

The second phase witnessed a decline in population density in the majority of HTNV-dominant cities. In contrast, most of SEOV-dominant cities were continuing to experience an increase in population density. The comparable patterns of built-up land were observed in both types of cities during the two study periods. Additionally, the per capita cropland area showed minimal changes in numerous HTNV-dominant cities during the two study phases, while in most SEOV-dominant cities, there was a gradual decline in cropland each year (Supplementary Table 4). By employing LASSO regression for variables selection, we determined the urbanization-related factors and their corresponding lag terms that were deemed necessary to be included in each model. Following thorough evaluation and comparison of models, XGBoost emerged as the superior algorithm in modelling the effects of urbanization-related factors on HFRS incidence for both genotypes (Supplementary Fig. 6). We presented the pooled exposure-response curves of eight variables that exhibited significant impacts on the HFRS incidence, and five of them were found to contribute to both the two genotype infections with distinct effects and lags, i.e., built-up land, population density, green land, real GDP and impervious surface (Fig. 3). Among them, built-up land (lag = 3) and real GDP (lag = 2) presented the highest relative contribution to HTNV and SEOV infections, respectively (Supplementary Fig. 7). The impact of nightlight index and park green land was found to be more pronounced in HTNV-dominant cities, while cropland, impervious surface, and floor space of commercialized buildings sold contributed more significantly to HFRS incidence in SEOV-dominant cities.

The HFRS incidence in both HTNV-dominant and SEOV-dominant cities was influenced by built-up land in a similar manner. Specifically, the incidence initially increased rapidly and then stabilized as the variable increased over lag periods of two to three years. There was a similar manner regarding the nightlight index (lag = 0) for HTNV-dominant cities and impervious surface (lag = 2) for SEOV-dominant ones (Fig. 3A, B, K and N). While the impact of impervious surface in HTNV-dominant cities exhibited a more pronounced decline in HFRS incidence when the impervious surface (lag = 0) increased to approximately 80 m^2^ per capita (Fig. 3I and J). In contrast, population density had a predominantly negative effect on the incidence of both the two major categories of endemicity, with longer lag period observed for SEOV-dominant cities (Fig. 3C and D). The associations between green land (lag = 3), cropland (lag = 3) and floor space of commercialized buildings sold (lag = 0) with HFRS incidence in SEOV-dominant cities displayed a first downward trend followed by an upward trend, which was similar to the effect of green land (lag = 3) on HFRS incidence in HTNV-dominant ones (Fig. 3E, F, L and P). Real GDP displayed opposite U-shaped patterns of effect on HFRS incidence in the two categorical cities (Fig. 3G and H). Additionally, the exposure-response curves of built-up land (lag = 0) and park green land (lag = 3) for HTNV-dominant cities showed fluctuating trends (Fig. 3M and O).

## 4 Discussion

China is currently facing the most severe threat from the HFRS epidemic worldwide. Despite previous evidence suggesting variations in geographic distribution between HTNV-dominant cities and SEOV-dominant cities, we present a comprehensive perspective highlighting difference in the distribution range, clinical manifestation, urbanization-related factors for the two distinct serotypes, as well as the conversion of regional endemic categories for HFRS. In the mainland of China, the *Apodemus agrarius*, as the primary animal host of HTNV, predominantly inhabits agricultural lands, shrublands, and grasslands, with a positive detection rate of HTNV ranging from 4.97 % to 68.75 % [13,14]. While the *Rattus norvegicus*, mainly carrying SEOV, prefers human residential areas such as houses, warehouses, and kitchens, with a positive detection rate of SEOV ranging from 8.24 % to 21.00 % [15,16]. A study has indicated that the habitat ranges of the animal hosts for HTNV and SEOV are expanding, potentially leading to changes in hantavirus distribution patterns [17].

The number of mixed endemic cities increased in most regions of the country from 2001 to 2010 to 2011–2020. In contrast, the numbers of HTNV-dominant cities and SEOV-dominant cities tended to grow in northern (e.g., Northeast, North and Northwest China) and southern (e.g., Southwest and South China) geographical regions, respectively. In the past decade, numerous cities in the northern China have been confronted with potential urban decay characterized by aging and outflow of populations, resource depletion [18,19]. Rural-urban human migration is taking place, potentially results in reduced forest exploitation and abandonment of marginal cropland [20]. In areas where mechanized farming is impractical due to rugged terrain, agriculture gradually becomes abandoned. Consequently, more land becomes available for forest restoration [21]. The natural habitat suitable for the *Apodemus agrarius* is expanding due to forest restoration, thereby contributing to an increased risk of HTNV infection and facilitating the shift of other endemic types of cities to HTNV-dominant ones [22]. However, the southern China is witnessing a significant influx of external population and rapid urban development [23]. As population density increases and urban expansion impacts on rodent habitats, opportunities for human-rodent interaction will rise, leading to an elevated risk of human infection with SEOV [24]. Meanwhile, urbanization has provided more suitable ecological niches for *Rattus norvegicus*, further promoting the population expansion of these SEOV carriers and the spread of SEOV among host animals. As revealed by our findings, SEOV infections are dominating the epidemic of HFRS in an increasing number of cities in the mainland of China, aligning with the global trends of HFRS in the epidemiology. Studies have confirmed that SEOV is continuously emerging in regions that were previously non-existent, facilitated by international trade [25], which may be closely related to fact that *Rattus norvegicus* can be transported to various parts of the world via different modes of transportation. China's well-developed transportation infrastructure has provided convenience for the migration of *Rattus norvegicus*, which may expose previously uninfected humans and animals to the SEOV, potentially leading to the emergence of new SEOV-dominant regions or the shift of original endemic types. Therefore, the characteristics of local urban development necessitate the formulation and implementation of targeted prevention and control measures for HFRS, as well as the adoption of effective deratization strategies.

The differences between the two genotypes were also noted in their clinical characteristics. Our modeling analysis indicate that patients of HFRS with gastrointestinal symptoms were more likely to be infected with SEOV. The findings have the potential in early genotype discrimination for HFRS patients based on clinical characteristics. This can enable intervention in advance before patients progress to more severe clinical outcomes resulting from HTNV infections [26].

China is currently undergoing the most extensive and rapid urbanization process globally. While urbanization has brought a lot of convenience, it has also brought about various challenges such as overcrowding of urban population, frequent changes in land use, and ecological environment degradation [27]. Moreover, by utilizing the XGBoost model, we have identified the distinct impact of urbanization-related factors on HFRS incidence separately in HTNV-dominant and SEOV-dominant cities. Overall, the influence of urbanization-related factors on HFRS incidence was found to be diverse both for the two major categories of endemicity. We observed a strong association between the incidence of HFRS and urban expansion-related factors, such as impervious surface and built-up land for both HTNV-dominant and SEOV-dominant cities. Additionally, we found a significant association between HFRS incidence and urban virescence-related factors, such as green land in both cities and park green land in HTNV-dominant cities. It has been suggested that the conversion of land cover types and fragmentation of biological habitat, can result in unprecedented mixing of wild rodent species and humans which can increase exposure to rodents carrying pathogens and elevate the risk of infection for diseases [28].

Previous studies have demonstrated that urbanization has significantly altered the biodiversity of urban landscapes [29,30], and the presence of urban green spaces in highly urbanized areas plays a crucial role in conserving biodiversity [31]. For instance, green spaces can provide suitable habitats and refuges for wild and commensal rodents [32]. Additionally, the increased opportunities for rodent interactions with outdoor residents may consequently elevate the risk of hantavirus infection [33,34]. However, as urban expansion and greening continue to develop, the process of urbanization will gradually be completed [35], resulting in overall improvements in the urban environment, sanitation conditions, epidemic prevention strategies, and population awareness of self-protection. Consequently, the incidence of HFRS may gradually stabilize and eventually decline [36,37]. These findings emphasize the need for heightened vigilance regarding the potential epidemic risk of HFRS in rapidly developing cities with strong construction efforts.

The limitations of this study should also be acknowledged. First, there is considerable variation in the quality of testing and reporting of hantavirus genotypes in rodents and HFRS patients across different cities. Consequently, some locations classified as pseudo-absence may represent false negatives, resulting in an underestimation of the model-estimated risk for the three endemic categories of HFRS. Second, our modeling analysis did not incorporate certain urbanization-related factors associated with HFRS incidence due to data unavailability, such as HFRS vaccine inoculation rates and public education efforts regarding HFRS [38,39], which could introduce bias into the estimation of relative contributions made by these determinants identified in our study. Therefore, future modeling efforts should expand their data sources to analyze the impact of these factors on HFRS incidence.

In conclusion, our study predicts the geographic distribution of three distinct endemic categories for HFRS, establishes a symptom-based model for predicting infected genotypes, and reveals the association between HFRS incidence of different hantavirus genotypes and urbanization-related factors. Determining the dominant genotypes in various cities and understanding the urbanized factors associated with incidence can offer a novel perspective for accurately predicting and early warning of HFRS. Our modeling results address a critical gap in the national landscape concerning the three endemic categories of HFRS, which may guide targeted surveillance development and prevention/control measures amidst rapid urbanization. In the current era of rapid urbanization, it is imperative to further enhance the surveillance for the spread of hantavirus. For newly emerging epidemic areas of hantavirus infections, it is crucial to intensify publicity efforts regarding HFRS, raise public awareness of HFRS prevention, and reinforce vaccination for susceptible populations. For the mixed endemic cities, attention should not only be paid to rodent prevention facilities, food safety, and personal protection in areas with frequent changes in the types of land use, but also to comprehensively improve residential living environments such as urban green land and park green land, thereby reducing the likelihood of contact between humans and rodents.

## CRediT authorship contribution statement

**Yao Tian:** Writing – original draft, Visualization, Software, Methodology, Data curation, Conceptualization. **Tao Wang:** Visualization, Software, Methodology. **Jin-Jin Chen:** Data curation. **Qiang Xu:** Data curation, Conceptualization. **Guo-Lin Wang:** Data curation, Conceptualization. **Bao-Gui Jiang:** Data curation, Conceptualization. **Li-Ping Wang:** Writing – review & editing, Supervision, Project administration, Methodology, Conceptualization. **Chen-Long Lv:** Writing – original draft, Visualization, Software, Data curation. **Tao Jiang:** Writing – review & editing, Supervision, Project administration, Methodology, Conceptualization. **Li-Qun Fang:** Writing – review & editing, Supervision, Project administration, Methodology, Conceptualization.

## Data availability

The relevant data of hantavirus genotypes and the model are publicly available and are provided in the supplementary information. Raw data on HFRS cases are not publicly available and are protected due to data privacy laws, which were used under license for the current study, but are available upon reasonable request to the corresponding author and with permission from the data provider (Li-Ping Wang).

## Ethics approval

This study did not require ethical approval because the data on laboratory- and clinically-confirmed cases of HFRS at the city level obtained from the CISDCP and the hantavirus genotypes identified in humans and rodents was compiled by integrating data from diverse sources such as literature review (PubMed, Web of Science, GenBank, China National Knowledge Infrastructure and WanFang database), the GenBank database, regular annual surveillance for selected notifiable infectious diseases, and the national etiology surveillance program for febrile hemorrhagic syndrome. All the original data were anonymous.

## Funding

This work was funded by 10.13039/501100012166National Key Research and Development Program of China (2023YFC2605603), the 10.13039/501100001809National Natural Science Foundation of China (82304208 to Chen-Long Lv) and the Fund of 10.13039/501100015976State Key Laboratory of Pathogen and Biosecurity (SKLPBS2205).

## Declaration of competing interest

The authors declare that they have no known competing financial interests or personal relationships that could have appeared to influence the work reported in this paper.
