# Supplementary material for: Distribution dynamics and urbanization-related factors of Hantaan and Seoul virus infections in China between 2001 and 2020: A machine learning modelling analysis
Source: Heliyon. 2024 Oct 29;10(21):e39852. doi: 10.1016/j.heliyon.2024.e39852 (PMC11566693; doi:10.1016/j.heliyon.2024.e39852)
Supplement: Multimedia component 1 [file mmc1.docx]

**Supplementary appendix**

Distribution dynamics and urbanization-related factors of Hantaan and Seoul virus infections in China between 2001 and 2020

**Table of Contents**

| **Page** | **Item** |
| --- | --- |
| 1−11 | **Supplementary Materials and Methods** |
| 12 | **Supplementary Reference 1** |
| 13−14 | **Supplementary Table 1.** The spatial resolution, study duration and source of the included data. |
| 15−19 | **Supplementary Table 2.** Variables used for modelling in this study. |
| 20 | **Supplementary Table 3.** Index of retrieved literatures for two hantavirus genotypes. |
| 21 | **Supplementary Table 4.** The annual trend changes of urbanization-related factors of HTNV-dominant and SEOV-dominant cities during different phases. |
| 22 | **Supplementary Figure 1.** The flow diagram of literature review. |
| 23 | **Supplementary Figure 2.** The observed geographical distributions of HTNV and SEOV during two ten-year intervals. |
| 24 | **Supplementary Figure 3.** Effects of major variables (top nine) for predicting the proportion of HTNV based on the two-stage XGBoost model from 2001 to 2010. |
| 25 | **Supplementary Figure 4.** Effects of major variables (top nine) for predicting the proportion of HTNV based on the two-stage XGBoost model from 2011 to 2020. |
| 26 | **Supplementary Figure 5.** Effects of major variables (top nine) indicated by SHAP values in the optimal modelling (XGBoost) for patients infected with HTNV and SEOV |
| 27 | **Supplementary Figure 6.** Comparison of modelling for the association of urbanization-related factors with HFRS incidence. |
| 28 | **Supplementary Figure 7.** Importance and effects of variables based on the XGBoost model for the association of urbanization-related factors with HFRS incidence. |
| 29−36 | **Supplementary Reference 2** |

**Supplementary Materials and Methods**

***Data from other sources***

The regular annual surveillance for HFRS and its animal hosts was initiated in 2005, leading to the establishment of a total of 41 surveillance sites across 22 provinces by 2020. From which the information on hantavirus genotypes detected from humans or rodents was included in this study.

Between January 2009 and December 2020, a nationwide active surveillance program, implemented by the Chinese Center for Disease Control and Prevention (China CDC), was administered on patients with febrile hemorrhagic syndrome that were recruited from 168 sentinel hospitals in the mainland of China [1]. The number of sentinel hospitals were determined in proportion to the total population size of the province, and the hospitals with high-coverage medical service, adequate capacities of surveillance and laboratory testing were chosen within each province. a total of 835 individual cases of HFRS, with available hantavirus genotyping and clinical information, were extracted from the national etiology surveillance program for febrile hemorrhagic syndrome. A standard operating procedure (SOP) that encompassed patient's enrollment, specimen's collection, laboratory testing, data recording and management, was developed by China CDC. All participating hospitals used the same protocol and provided training for personnel before the implementation of the project. Febrile hemorrhagic syndrome was defined as acute fever (temperature ≥ 37.5°C) of less than three weeks, with two or more clinical manifestations: 1) cutaneous hemorrhage; 2) mucosal hemorrhage; 3) nasal cavity hemorrhage; 4) hemoptysis; 5) hematemesis; 6) hematochezia; 7) anemia; 8) thrombocytopenia; 9) other hemorrhage manifestations [2]. Those patients who were not initially diagnosed in sentinel hospitals and referred from other hospitals, were excluded from the current study. For each recruited patient, individual information on demography (sex, age, residing address), clinical manifestations (detailed signs/symptoms and the onset date, etc.), information on laboratory testing and medication usage were collected by a standardized case reporting form (CRF) and entered into a standardized database by trained clinicians.

For each eligible patient included in the program, one or more types of specimens, including blood, cerebrospinal fluid, secretion, urine, and lymphatic fluid, were collected following the SOP depending on the clinical manifestations. All samples should be sent to the laboratory within a 24-h period at 4°C−8°C and tested immediately, and if not, were stored at -80 °C until tested. The testing and genotyping for hantavirus was performed by RT-PCR assay of blood samples, and the main principle was to sequence viral RNA strains from blood samples, reverse-transcribe it to cDNA, and perform amplification of DNA using appropriate typing primers. After identification by agarose gel electrophoresis, nucleic acid sequence analysis was performed on the PCR-amplified positive samples, and the virus genotype was determined after the sequence comparison [3]. According to the SOP, the experimental procedure contained 6 steps: 1) extracting RNA from blood samples by using different methods (QIAamp Viral RNA Mini kit, RNeasy Mini Kit, Trizol method); 2) synthetizing cDNA by using the SuperScript III RT Reverse Transcription System; 3) nested-PCR analysis assay; 4) RT-PCR nucleic acid assay; 5) agarose gel electrophoresis analysis; 6) nucleic acid sequence analysis [2].

In addition to hantavirus, the collected samples were also screened for 11 species of febrile hemorrhagic syndrome related pathogens, including six species of bacteria (*Leptospira*, *Streptococcus suis*, *Yersinia pestis*, *Anaplasma phagocytophilum*, *Ehrlichia chaffeensis*, *Rickettsia*), four species of viruses (Dengue virus, Crimean-Congo hemorrhagic fever virus, Severe fever with thrombocytopenia syndrome virus, Ebola virus), and one species of parasite (*Babesia*). The National Health Commission of the People’s Republic of China decided that since data from patients with febrile hemorrhagic syndrome was part of continuing public health surveillance and implemented national surveillance guidelines; parents/guardians of participants in this program were only required to provide brief verbal consent during their enrollment, which was recorded in each questionnaire by their physicians. This project and the procedure for obtaining consent were approved by the ethical review committee of China CDC (2015-025).

***Data extraction and processing***

Data on 11 environmental, 19 bioclimatic, 10 biological, and 10 urbanization-related variables potentially associated with geographic distribution of hantavirus genotypes were collected and processed at the city level. Among them, the raster-map lays of environmental factors at the city level included annual average normalized difference vegetation index (NDVI) with a spatial resolution of 1 km that comprehensively reflects the vegetation growth in an area and 2000 elevation with a spatial resolution of 1 km which were collected from Resource and Environment Science and Data Center (<https://www.resdc.cn/>) [4], as well as land cover with nine categories with a spatial resolution of 30 m which were provided by Yang et al [5]. The raster-map lays of annual bioclimatic indicators with a spatial resolution of 25 arcminutes, including BIO1‒BIO19, were provided by WorldClim (http://www.worldclim.org). The raster-map lays of biological factors at the city level included 2010 livestock density, 2013 mammalian richness and 2018 rodent richness. Eight kinds of livestock density, including buffalo, cattle, goat, sheep, horse, pig, duck and chicken with a spatial resolution of 1 km, were collected from Food and Agriculture Organization (http://www.fao.org/livestock-systems/en/). The mammalian richness with a spatial resolution of 30 arcseconds was collected from International Union for Conservation of Nature (https://sedac.ciesin.columbia.edu/). The rodent richness with a spatial resolution of 1 km was collected from BiodiveristyMapping.org (https://biodiversitymapping.org/). The urbanization-related data at the city level, including four social-demographic (real gross domestic product [GDP], population density, nightlight index and electricity consumption), four urban built-up (land used for urban construction, built-up land, real estate investment and floor space of commercialized buildings sold) and two urban landscape (park green land and green land) variables, were extracted from National and Local Bureau of Statistics. The representative value for explanatory variables with multiple annual data was determined by calculating the average for each city within each time phase, while four variables (elevation, livestock density, mammalian richness and rodent richness) were incorporated in the modelling analysis using the most recent available year-specific data.

All the raster-type map lays were overlapped on the city-level vector digital map of China and these factors were calculated for the average and summation by using the zonal statistical calculation technique. The calculation and process on raster type data were implemented by using ArcGIS 10.7 (Environmental Systems Research Institute Inc., Redlands, CA, USA).

***Inclusion of factors related to urbanization***

We have incorporated 13 factors closely related to urbanization, some of which have already been applied in ecological modelling for HFRS or rodents (Supplementary Table 2). Previous studies have demonstrated that the land used for urban construction, park green space, green areas, and built-up land can significantly influence the abundance, diversity, community composition, habitat conditions, and food acquisition pattern of rodents, thereby affecting the contact opportunities between humans and rodents while altering the exposure risk associated with hantavirus. Factors such as real GDP, population density, nightlight index, electricity consumption, and real estate investment, which are closely associated with the level of urbanization development, may influence the likelihood of people being infected with hantavirus by affecting local hygiene conditions, access to education and healthcare, as well as public awareness of prevention. Therefore, owing to the direct or indirect effects of these factors on the risk of hantavirus infection, we included these urbanization-related factors and analyzed their association with the HFRS incidence.

***Extreme gradient boosting (XGBoost) model***

The XGBoost algorithm is an highly efficient machine learning technique that builds a series of decision trees to improve predictive performance, based on the gradient boosting framework proposed by Chen et al. in 2016 [6]. In comparison to traditional machine learning models, XGBoost demonstrates faster computational speed and superior predictive accuracy for small to medium sized datasets due to its modified learning method and incorporation of regularization techniques. Moreover, XGBoost employs regularization techniques to prevent overfitting and can automatically handle missing values. Additionally, it supports a variety of objective functions and evaluation metrics, making it suitable for a wide range of classification and regression problems. Chen et al. utilized XGBoost and SHAP to quantify influencing factors and their effects on interactions among influenza types/subtypes [7]. Man et al. employed SARIMA-XGBoost model to explore the risk factors of hand foot and mouth disease (HFMD) [8]. In our study, a two-stage XGBoost model was conducted to predict the potential endemic types (HTNV-dominant, SEOV-dominant, and mixed endemic cities) for cities without available genotyping information in each phase. The objective function of the XGBoost model algorithm is:

${Obj}_{m}=\sum_{i=1}^{n} l((y_{i}, {\hat{y}_{i}}^{m-1})+f_{m}(x_{i}))+\Omega(f_{m})$ (1)

where $n$ represents the total number of records for all the included cities during the corresponding time span. $y_{i}$ represents the observed value of the dependent variable for city $i$ in a given year. $m$ represents the number of iterations. ${\hat{y}_{i}}^{m-1}$ represents the sum of the predicted values of city $i$ in a given year for the previous $m-1$ iterations. $f_{m}(x_{i})$ represents the predicted value of the dependent variable at the new tree calculated by all independent variables $x_{i}$ for cities $i$ in a given year. $f_{m}$ represents the error in the $m$ iterations. $l$ represents the cost function, which is used to measure the difference between the label and the prediction in the last step, as well as the output of the new tree, and $\Omega$ is the regularisation term that punishes the complexity of the new tree. The cost function is extended to a second-order Taylor expansion, and L1 and L2 regularisation are introduced at the same time to avoid overfitting.

***Shapley additive explanations (SHAP)***

SHAP is a method to enhance the interpretability of models based on coalitional game theory, with the Shapley values for each feature (usually referred to independent variable) calculated by estimating the average contribution of all the observation for this feature to the response variable [9]. SHAP estimates how much information we would earn or lose in the presence or absence of a particular feature to determine the contribution of each feature in terms of prediction. To obtain the Shapley value for a particular feature, a weighted sum of the differences between the information in the presence and absence of the feature is calculated for each observation. SHAP is an additive interpretation model based on an explanation model *g*, defined as follow:

$g\left( z^{'} \right)=\emptyset_{0}+\sum_{p=1}^{M} \emptyset_{p}$ (1)

where $\emptyset_{p}$ is the $p$-th feature’s Shapley value, and *M* is the number of features. $\emptyset_{0}$ is a constant.

To calculate $\emptyset_{p}$, we first calculated the model output for each subset of features with characteristic order of presence or absence of feature $p$, and calculated the product of the difference between the two and the probability of presence of the subset. The marginal contribution of feature $p$ under this subset was then calculated and the marginal contributions of all subsets were weighted to sum up to obtain the Shapley value of feature $p$. The positive and negative Shapley values represent whether the feature has a positive or negative effect on the model, respectively.

***Model development of two-stage XGBoost***

In the two-stage XGBoost modelling of geographic distribution for different hantavirus genotypes, the representative value for explanatory variables with multiple annual data was determined by calculating the average for each city within each time phase, while four variables (elevation, livestock density, mammalian richness and rodent richness) were incorporated in the modelling analysis using the most recent available year-specific data. The variance inflation factor (VIF) was used to screen variables to mitigate multicollinearity, and only variables with VIF less than 10 were retained. Subsequently, we built a pre-model for each of the two-stage XGBoost models by including all the variables filtered by the multicollinearity test to test the performance of the models using 10-fold cross-validation. The two-stage model was separately fitted 100 times to predict the probability and proportion of occurrence for each hantavirus genotype to identify HTNV-dominant, SEOV-dominant and mixed endemic areas.

The initial stage of the modelling analysis involved a XGBoost model with a “logistic” structure to predict cities at risk of HTNV or SEOV occurrence, respectively. HTNV-dominant (or SEOV-dominant) cities with available genotyping data were considered as “cases”, and cities without HFRS reported cases and only with SEOV (or HTNV) occurrence as “controls”. A probability of HTNV (or SEOV) occurrence was projected for each city by this stage. In the second stage, we constructed a XGBoost model with a “squaredlogerror” structure to further differentiate between cities at risk of hantavirus occurrence that are predominantly by HTNV, SEOV, or both hantaviruses. The projected probabilities of HTNV and SEOV occurrences from the initial stage model were included in the second stage analysis as two additional predictors. The proportion of reported HTNV occurrence in cities with available genotyping data was taken as response variable for the two study phases. Based on this, the projected proportion of HTNV occurrence was estimated for all cities that have reported hantavirus occurrence and those identified at risk of HTNV or SEOV occurrence during the initial stage modelling analysis. Subsequently, these cities were classified into HTNV-dominant (projected proportion of HTNV≥80%), SEOV-dominant (projected proportion of HTNV≤20%), and mixed endemic cities (projected proportion of HTNV between 20% to 80%) for the two study phases, respectively, which were mapped and the transition between endemic types was illustrated using a Sankey diagram.

In addition to 10-fold cross-validation, we utilized hantavirus genotyping data spanning from 2011‒2015 and 2006‒2010 as external data to validate the two-stage extreme gradient boosting (XGBoost) models constructed in Phase I (2001‒2010) and Phase II (2011‒2020), respectively. Specifically, we selected 50 cities with over 10 cumulative detection records of hantavirus genotypes during 2011‒2015 (or 2006‒2010), and categorized each city as HTNV-dominant (proportion of HTNV≥80%), or SEOV-dominant (proportion of HTNV≤20%), or mixed endemic one (proportion of HTNV between 20% to 80%) based on the proportion of HTNV detection records. On the other hand, we used the model established in Phase I (or II) to project the proportions of HTNV for these cities, based on which we redefined the endemic type for each city using the same criteria. The predictive performance of model was validated by comparing the endemic type determined through genotyping data with that projected by models for each city. The XGBoost model was implemented using the R packages (version 4.2.1) “xgboost” and “ParBayesianOptimization”

***Random forest (RF)***

RF is another classical ensemble learning model widely used [10]. The training algorithm of RF is based on bootstrap aggregating. Each tree is trained on many bootstrap samples, and was then evaluated using the remaining data to produce more accurate predictions. The unknown value of an observation will be calculated by the majority vote of the out-of-bag predictions for that observation [11]. This approach reduces the risk of overfitting, which can occur with individual decision trees, by introducing randomness in both feature selection and data sampling. RF not only provides high accuracy but also estimates feature importance, which helps in understanding which features are most critical for the predictions. It is easy to use and well-suited for high-dimensional data. Andraud et al. utilized RF to identify the risk factors of African swine fever outbreak in Romania [12]. We optimized the key learning parameter, mtry, which defines the number of variables randomly sampled as candidates at each split, between the range of 10–30 using Bayesian parameter optimization algorithm, with a 10-fold cross-validation process to avoid overfitting. The R packages “ParBayesianOptimization” and “randomForest” were used to develop the RF model.

***Gradient boosting machine (GBM)***

GBM is a versatile ensemble learning technique that integrates gradient-based optimization and boosting techniques. Gradient-based optimization employs gradient computations to minimize the loss function of a model with respect to the training data. Boosting, on the other hand, employs an iterative process of assembling “weak learners” to create a robust predictive model suitable for regression and classification tasks [13]. The algorithm also performs automatic feature selection, giving priority to important variables while eliminating those that are deemed irrelevant or redundant. GBM can optimize different loss functions, making it very effective for solving complex non-linear problems. Its advantage lies in its ability to capture intricate interactions between features, leading to very high predictive accuracy. Azizi et al. used GBM for building prediction models to predict COVID-19 test positivity and explore the risk factors [14].We optimized the key learning parameter, interaction.depth and n.minobsinnode, separately between the range of 2–15 and 10-30 using Bayesian parameter optimization algorithm, with a 10-fold cross-validation process to avoid overfitting. The R packages “ParBayesianOptimization” and “gbm” were used to develop the GBM model.

***Generalized linear model (GLM)***

GLMs are a class of widely used statistical models that relate the expected value of the response variable to a linear combination of one or more predictor variables through a link function, which have been applied quite effectively in the modelling of a mean response under nonstandard conditions, where discrete as well as continuous data distributions can be accommodated [15]. GLMs can handle different types of response variables, such as binary variable (logistic regression) and count variable (Poisson regression). The strength of GLMs lies in their simplicity, interpretability, and solid statistical foundation, which allows them to be used for hypothesis testing and confidence interval construction. Charnley et al. explored the relationships between drought and cholera in Africa using GLMs [16].We first constructed a GLM with a Poisson structure, and then applied stepwise regressions based on the Akaike Information Criterion (AIC) to select the model with better fit and input features. The R packages “stats” was used to develop the GLM.

***Model evaluation and comparison***

In the modelling for differentiation of patients infected with HTNV and SEOV based on clinical characteristics, we compared the performance of four different modelling algorithms (XGBoost model, RF, GBM and GLM), through seven evaluating indicators including the AUC of the receiver operating characteristic (ROC) curve, AUC of the precision-recall (PR) curve, accuracy, sensitivity, specificity, F1 score and kappa coefficient.

As for the modelling of relationships between urbanization-related factors and HFRS incidence of different endemic categories, we constructed four modelling algorithms the same as above. Differently, due to using continuous variable as outcome here in these models, we assessed their performance through other five evaluating indicators including Pearson correlation coefficient, root mean squared error (RMSE), mean absolute error (MAE), normalized root mean squared error (NRMSE) and root mean squared logarithmic error (RMSLE). Except for RMSE, MAE, NRMSE and RMSLE, the higher values of all other evaluating indicators represent the better fitting of the model, and we comprehensively considered the ranking of all corresponding indicators to separately determine the best algorithm. After screening the optimal models the autocorrelation term for each city’s incidence in the previous year was determined using the autocorrelation function (ACF) from the R package "tseries". In addition to assessing the association between HFRS incidence and each independent variable in the same year (indicated by lag = 0), we also considered the delayed effect of these urbanization-related variables, which were computed as a moving average for both the current year and the previous 1‒3 years. The lagged effect of ecoclimatic covariates was exclusively considered within the preceding one year. The missing values of individual time points for the independent variables were imputed using the Kalman filter method via the R package "imputeTS". Least absolute shrinkage and selection operator (LASSO) regression analysis was conducted to select the inclusion of urbanization-related variables and their associated lag terms using the R package "glmnet", while keeping the ecoclimatic covariates and autocorrelation term fixed in the model.

References 1

[1] Management Office of National Science and Technology Major Project of China CCfDCaP. Febrile Hemorrhagic Syndrome Surveillance Protocol. **2019**.

[2] Wei L, Zhuo Z. Pathogen Surveillance and Detection Techniques: Febrile Hemorrhagic Syndrome SUN YAT-SEN UNIVERSITY PRESS, 2019.

[3] Bagamian KH, et al. Transmission ecology of Sin Nombre hantavirus in naturally infected North American deermouse populations in outdoor enclosures, PLoS One 7 (10) (2012) e47731. <https://doi.org/10.1371/journal.pone.0047731>

[4] Xu X. Spatial Distribution Data Set of 1km-resolution Annual NDVI in China (1998-2015), Upper Yangtze River Scientific Data Center (2022). <https://doi.org/DOI>: 10.12078/2018060601

[5] Yang J, Huang X. The 30m annual land cover dataset and its dynamics in China from 1990 to 2019, Earth Syst Sci Data 13 (8) (2021) 3907-25. <https://doi.org/10.5194/essd-13-3907-2021>

[6] Chen T, Guestrin C. XGBoost: A Scalable Tree Boosting System, Proceedings of the 22nd ACM SIGKDD International Conference on Knowledge Discovery and Data Mining (2016).

[7] Chen Y, et al. Global pattern and determinant for interaction of seasonal influenza viruses, J Infect Public Health 17 (6) (2024) 1086-94. <https://doi.org/10.1016/j.jiph.2024.04.024>

[8] Man H, et al. Analysis of a SARIMA-XGBoost model for hand, foot, and mouth disease in Xinjiang, China, Epidemiol Infect 151 (2023) e200. <https://doi.org/10.1017/s0950268823001905>

[9] Lundberg SM, Lee S-I. A Unified Approach to Interpreting Model Predictions, ArXiv abs/1705.07874 (2017).

[10] Dunne R, et al. Thresholding Gini variable importance with a single-trained random forest: An empirical Bayes approach, Comput Struct Biotechnol J 21 (2023) 4354-60. <https://doi.org/10.1016/j.csbj.2023.08.033>

[11] Zhang J, et al. Risk prediction of two types of potential snail habitats in Anhui Province of China: Model-based approaches, PLoS Negl Trop Dis 14 (4) (2020) e0008178. <https://doi.org/10.1371/journal.pntd.0008178>

[12] Andraud M, et al. Spatiotemporal clustering and Random Forest models to identify risk factors of African swine fever outbreak in Romania in 2018-2019, Sci Rep 11 (1) (2021) 2098. <https://doi.org/10.1038/s41598-021-81329-x>

[13] Hastie T, et al. The Elements of Statistical Learning: Data Mining, Inference, and Prediction, Math Intell 27 (2004) 83-5. <https://doi.org/10.1007/BF02985802>

[14] Azizi Z, et al. Importance of sex and gender factors for COVID-19 infection and hospitalisation: a sex-stratified analysis using machine learning in UK Biobank data, BMJ Open 12 (5) (2022) e050450. <https://doi.org/10.1136/bmjopen-2021-050450>

[15] Khuri A, et al. Design Issues for Generalized Linear Models: A Review, Stat Sci 21 (2007). <https://doi.org/10.1214/088342306000000105>

[16] Charnley GEC, et al. Exploring relationships between drought and epidemic cholera in Africa using generalised linear models, BMC Infect Dis 21 (1) (2021) 1177. <https://doi.org/10.1186/s12879-021-06856-4>

**Supplementary Table 1. The spatial resolution, study duration and source of the included data.**

| **Type** | **Data** | **Spatial resolution** | **Study duration** | **Source of data** | **Website** | **Reference** |
| --- | --- | --- | --- | --- | --- | --- |
| Biological factors | Livestock density | 1 km | 2010 | Food and Agriculture Organization (FAO) | http://www.fao.org/livestock-systems/en/ | Gilbert M, Nicolas G, Cinardi G, et al. Global distribution data for cattle, buffaloes, horses, sheep, goats, pigs, chickens and ducks in 2010. *Sci Data*, 2018; 5: 180227. |
|  | Mammalian richness | 0° 0′ 30″ | 2013 | International Union for Conservation of Nature (IUCN) | https://sedac.ciesin.columbia.edu/ | International Union for Conservation of Nature - IUCN, and Center for International Earth Science Information Network - CIESIN - Columbia University. 2015. Gridded Species Distribution: Global Mammal Richness Grids, 2015 Release. Palisades, NY: NASA Socioeconomic Data and Applications Center (SEDAC). |
|  | Rodent richness | 10 km | 2018 | BiodiversityMapping.org | https://biodiversitymapping.org/ | Jenkins, C.N. & K. Van Houtan. (2016). Global and regional priorities for marine biodiversity protection. Pimm, SL, CN Jenkins, R Abell, TM Brooks, JL Gittleman, LN Joppa, PH Raven, CM Roberts, JO Sexton (2014) The biodiversity of species and their rates of extinction, distribution, and protection. *Science* 344(6187): 1246752. |
| Ecoclimatic factors | Climate data (19 variables) | 0° 25′ | 1975‒2018 | WorldClim | https://www.worldclim.org/ | 1. Fick SE, Hijmans RJ. WorldClim 2: new 1-km spatial resolution climate surfaces for global land areas. Int. J. Climatol., 2017; 37: 4302-15. 2. Harris I, Jones PD, Osborn TJ, Lister DH. Updated high-resolution grids of monthly climatic observations – the CRU TS3.10 Dataset. *Int. J. Climatol*., 2014; 34: 623-42. |
| Environmental factors | Land cover (9 variables) | 30 m | 1990‒2021 | Earth System Science Data | https://zenodo.org/ | Yang, J. & Huang X. The 30 m annual land cover dataset and its dynamics in China from 1990 to 2019. Earth *Syst. Sci. Data*, 2021: 13(8):3907-3925. |
|  | NDVI | 1 km | 1998‒2019 | Resource and Environment Science and Data Center | https://www.resdc.cn/ | Annual Normalized Difference Vegetation Index (NDVI) spatial distribution dataset in China. |
|  | Elevation | 1 km | 2000 | Resource and Environment Science and Data Center | https://www.resdc.cn/ | Shuttle Radar Topography Mission (SRTM) |
| Urbanization-related factors | Land used for urban construction | Annual data at the city level. | 2001‒2019 | National Bureau of Statistics | https://www.stats.gov.cn/english/ | National and Local Bureau of Statistics |
|  | Park green land | Annual data at the city level. | 2001‒2019 | National Bureau of Statistics | https://www.stats.gov.cn/english/ | National and Local Bureau of Statistics |
|  | Green land | Annual data at the city level. | 2001‒2019 | National Bureau of Statistics | https://www.stats.gov.cn/english/ | National and Local Bureau of Statistics |
|  | Built-up land | Annual data at the city level. | 2001‒2019 | National Bureau of Statistics | https://www.stats.gov.cn/english/ | National and Local Bureau of Statistics |
|  | Real GDP | 1 km | 1992‒2019 | Scientific Data | https://doi.org/10.6084/m9.figshare.17004523.v1/ | Chen J, Gao M, Cheng S, et al. Global 1 km × 1 km gridded revised real gross domestic product and electricity consumption during 1992-2019 based on calibrated nighttime light data. *Sci Data*. 2022; 9(1): 202. |
|  | Population density | 1 km | 2000‒2020 | Worldpop | https://www.worldpop.org/ | WorldPop and Center for International Earth Science Information Network (CIESIN), Columbia University (2018). Global High Resolution Population Denominators Project - Funded by The Bill and Melinda Gates Foundation (OPP1134076). https://dx.doi.org/10.5258/SOTON/WP00674 |
|  | Nightlight index | 1 km | 1992‒2021 | IEEE Transactions on Geoscience and Remote Sensing | https://dataverse.harvard.edu/dataset.xhtml?persistentId=doi:10.7910/DVN/GIYGJU/ | Wu Y, Shi K, Chen Z, Liu S, Chang Z, Developing Improved Time-Series DMSP-OLS-Like Data (1992–2019) in China by Integrating DMSP-OLS and SNPP-VIIRS, *IEEE Transactions on Geoscience and Remote Sensing*, 2022; 60: 1-14 |
|  | Electricity consumption | 1 km | 1992‒2019 | Scientific Data | https://doi.org/10.6084/m9.figshare.17004523.v1/ | Chen J, Gao M, Cheng S, et al. Global 1 km × 1 km gridded revised real gross domestic product and electricity consumption during 1992-2019 based on calibrated nighttime light data. *Sci Data*. 2022;9(1): 202. |
|  | Real estate investment | Annual data at the city level. | 2001‒2019 | National Bureau of Statistics | https://www.stats.gov.cn/english/ | National and Local Bureau of Statistics |
|  | Floor space of commercialized buildings sold | Annual data at the city level. | 2001‒2019 | CEIC Data Company Limited | https://www.ceicdata.com/ | China Premium Database |

NDVI: normalized difference vegetation index. GDP: gross domestic product.

**Supplementary Table 2.** **Variables used for modelling in this study.**

| **Data** | **Variables** | **Description** | **Reference** | **Usage and/or result** | **Missing data** |
| --- | --- | --- | --- | --- | --- |
| **Biological factors** | | | | |  |
| Livestock density | Buffalo | Density of buffalo (heads per km²) | Keesing F. Impacts of ungulates on the demography and diversity of small mammals in central Kenya. *Oecologia*. 1998;116(3):381-389. | Small mammal community maintained relatively constant species diversity on the plots to which ungulates did not have access | No missing data |
|  | Cattle | Density of cattle (heads per km²) | Davidson AD, Ponce E, Lightfoot DC, et al. Rapid response of a grassland ecosystem to an experimental manipulation of a keystone rodent and domestic livestock. *Ecology.* 2010;91(11):3189-3200. | Two different functional groups of herbivorous mammals, burrowing mammals and domestic cattle, have distinctive and synergistic impacts in shaping the structure and function of grassland ecosystems. | No missing data |
|  | Goat | Density of goat (heads per km²) | Shrestha R, McKenzie JS, Gautam M, et al. Determinants of clinical leptospirosis in Nepal. *Zoonoses Public Health*. 2018;65(8):972-983. | Goat owners were more likely to be infected by leptospirosis | No missing data |
|  | Sheep | Density of sheep (heads per km²) | Li G, Yin B, Wan X, et al. Successive sheep grazing reduces population density of Brandt's voles in steppe grassland by altering food resources: a large manipulative experiment. *Oecologia*. 2016;180(1):149-159. | Sheep grazing reduces population density of Brandt’s voles | No missing data |
|  | Horse | Density of horse (heads per km²) | Ward-Fear G, Brown GP, Pearson D, Shine R. Untangling the influence of biotic and abiotic factors on habitat selection by a tropical rodent. *Sci Rep*. 2021;11(1):12895. | Ingress of horses reduces population density of pale field rat | No missing data |
|  | Pig | Density of pig (heads per km²) | Liang W, Gu X, Li X, et al. Mapping the epidemic changes and risks of hemorrhagic fever with renal syndrome in Shaanxi Province, China, 2005-2016. *Sci Rep*. 2018;8(1):749. | Used in HFRS ecological modeling | No missing data |
|  | Duck | Density of duck (heads per km²) | Yin JX, Geater A, Chongsuvivatwong V, et al. Predictors for presence and abundance of small mammals in households of villages endemic for commensal rodent plague in Yunnan Province, China. *BMC Ecol*. 2008; 8:18. | Used in ecological modeling of small mammals | No missing data |
|  | Chicken | Density of chicken (heads per km²) | Yin JX, Geater A, Chongsuvivatwong V, Dong XQ, Du CH, Zhong YH. Predictors for abundance of host flea and floor flea in households of villages with endemic commensal rodent plague, Yunnan Province, China. *PLoS Negl Trop Dis*. 2011;5(3): e997. | Used in ecological modeling of flea | No missing data |
| Mammalian richness* | Mammalian richness | Number of mammal species per km² | Lovera R, Fernández MS, Jacob J, et al. Intrinsic and extrinsic factors related to pathogen infection in wild small mammals in intensive milk cattle and swine production systems. *PLoS Negl Trop Dis*. 2017;11(6): e0005722. | Pathogen infections increased with mammalian richness | No missing data |
| Rodent richness* | Rodent richness | Number of rodent species per km² | Xiao H, Lin X, Gao L, et al. Ecology and geography of hemorrhagic fever with renal syndrome in Changsha, China. *BMC Infect Dis*. 2013; 13:305. | Used in HFRS ENMs model | No missing data |
| **Ecoclimatic factors** | | | | |  |
| Climate data | BIO1 | Annual mean temperature (℃) | Andreo V, Glass G, Shields T, et al. Modeling potential distribution of Oligoryzomys longicaudatus, the Andes virus (Genus: Hantavirus) reservoir, in Argentina. *Ecohealth*. 2011;8(3):332-348. | Used in ecological modeling at the reservoir of Andes virus | No missing data |
|  | BIO2 | Mean diurnal range (mean of monthly [max temp-min temp]) (℃) | Andreo V, Glass G, Shields T, et al. Modeling potential distribution of Oligoryzomys longicaudatus, the Andes virus (Genus: Hantavirus) reservoir, in Argentina. *Ecohealth*. 2011;8(3):332-348. | Used in ecological modeling at the reservoir of Andes virus | No missing data |
|  | BIO3 | Isothermality (BIO2 / BIO7) (*100) | Andreo V, Glass G, Shields T, et al. Modeling potential distribution of Oligoryzomys longicaudatus, the Andes virus (Genus: Hantavirus) reservoir, in Argentina. *Ecohealth*. 2011;8(3):332-348. | Used in ecological modeling at the reservoir of Andes virus | No missing data |
|  | BIO4 | Temperature seasonality (standard deviation*100) | Andreo V, Glass G, Shields T, et al. Modeling potential distribution of Oligoryzomys longicaudatus, the Andes virus (Genus: Hantavirus) reservoir, in Argentina. *Ecohealth*. 2011;8(3):332-348. | Used in ecological modeling at the reservoir of Andes virus | No missing data |
|  | BIO5 | Max temperature of warmest month (℃) | Andreo V, Glass G, Shields T, et al. Modeling potential distribution of Oligoryzomys longicaudatus, the Andes virus (Genus: Hantavirus) reservoir, in Argentina. *Ecohealth*. 2011;8(3):332-348. | Used in ecological modeling at the reservoir of Andes virus | No missing data |
|  | BIO6 | Min temperature of coldest month (℃) | Andreo V, Glass G, Shields T, et al. Modeling potential distribution of Oligoryzomys longicaudatus, the Andes virus (Genus: Hantavirus) reservoir, in Argentina. *Ecohealth*. 2011;8(3):332-348. | Used in ecological modeling at the reservoir of Andes virus | No missing data |
|  | BIO7 | Annual range of temperature (BIO5- BIO6) (℃) | Andreo V, Glass G, Shields T, et al. Modeling potential distribution of Oligoryzomys longicaudatus, the Andes virus (Genus: Hantavirus) reservoir, in Argentina. *Ecohealth*. 2011;8(3):332-348. | Used in ecological modeling at the reservoir of Andes virus | No missing data |
|  | BIO8 | Mean temperature of wettest quarter (℃) | Andreo V, Glass G, Shields T, et al. Modeling potential distribution of Oligoryzomys longicaudatus, the Andes virus (Genus: Hantavirus) reservoir, in Argentina. *Ecohealth*. 2011;8(3):332-348. | Used in ecological modeling at the reservoir of Andes virus | No missing data |
|  | BIO9 | Mean temperature of driest quarter (℃) | Andreo V, Glass G, Shields T, et al. Modeling potential distribution of Oligoryzomys longicaudatus, the Andes virus (Genus: Hantavirus) reservoir, in Argentina. *Ecohealth*. 2011;8(3):332-348. | Used in ecological modeling at the reservoir of Andes virus | No missing data |
|  | BIO10 | Mean temperature of warmest quarter (℃) | Andreo V, Glass G, Shields T, et al. Modeling potential distribution of Oligoryzomys longicaudatus, the Andes virus (Genus: Hantavirus) reservoir, in Argentina. *Ecohealth*. 2011;8(3):332-348. | Used in ecological modeling at the reservoir of Andes virus | No missing data |
|  | BIO11 | Mean temperature of coldest quarter (℃) | Andreo V, Glass G, Shields T, et al. Modeling potential distribution of Oligoryzomys longicaudatus, the Andes virus (Genus: Hantavirus) reservoir, in Argentina. *Ecohealth*. 2011;8(3):332-348. | Used in ecological modeling at the reservoir of Andes virus | No missing data |
|  | BIO12 | Annual precipitation (mm) | Andreo V, Glass G, Shields T, et al. Modeling potential distribution of Oligoryzomys longicaudatus, the Andes virus (Genus: Hantavirus) reservoir, in Argentina. *Ecohealth*. 2011;8(3):332-348. | Used in ecological modeling at the reservoir of Andes virus | No missing data |
|  | BIO13 | Precipitation of wettest month (mm) | Andreo V, Glass G, Shields T, et al. Modeling potential distribution of Oligoryzomys longicaudatus, the Andes virus (Genus: Hantavirus) reservoir, in Argentina. *Ecohealth*. 2011;8(3):332-348. | Used in ecological modeling at the reservoir of Andes virus | No missing data |
|  | BIO14 | Precipitation of driest month (mm) | Andreo V, Glass G, Shields T, et al. Modeling potential distribution of Oligoryzomys longicaudatus, the Andes virus (Genus: Hantavirus) reservoir, in Argentina. *Ecohealth*. 2011;8(3):332-348. | Used in ecological modeling at the reservoir of Andes virus | No missing data |
|  | BIO15 | Precipitation seasonality (coefficient of variation) | Andreo V, Glass G, Shields T, et al. Modeling potential distribution of Oligoryzomys longicaudatus, the Andes virus (Genus: Hantavirus) reservoir, in Argentina. *Ecohealth*. 2011;8(3):332-348. | Used in ecological modeling at the reservoir of Andes virus | No missing data |
|  | BIO16 | Precipitation of wettest quarter (mm) | Andreo V, Glass G, Shields T, et al. Modeling potential distribution of Oligoryzomys longicaudatus, the Andes virus (Genus: Hantavirus) reservoir, in Argentina. *Ecohealth*. 2011;8(3):332-348. | Used in ecological modeling at the reservoir of Andes virus | No missing data |
|  | BIO17 | Precipitation of driest quarter (mm) | Andreo V, Glass G, Shields T, et al. Modeling potential distribution of Oligoryzomys longicaudatus, the Andes virus (Genus: Hantavirus) reservoir, in Argentina. *Ecohealth*. 2011;8(3):332-348. | Used in ecological modeling at the reservoir of Andes virus | No missing data |
|  | BIO18 | Precipitation of warmest quarter (mm) | Andreo V, Glass G, Shields T, et al. Modeling potential distribution of Oligoryzomys longicaudatus, the Andes virus (Genus: Hantavirus) reservoir, in Argentina. *Ecohealth*. 2011;8(3):332-348. | Used in ecological modeling at the reservoir of Andes virus | No missing data |
|  | BIO19 | Precipitation of coldest quarter (mm) | Andreo V, Glass G, Shields T, et al. Modeling potential distribution of Oligoryzomys longicaudatus, the Andes virus (Genus: Hantavirus) reservoir, in Argentina. *Ecohealth*. 2011;8(3):332-348. | Used in ecological modeling at the reservoir of Andes virus | No missing data |
| **Environmental factors** | | | | |  |
| Land cover | Cropland | Area of cropland (m² per capita) | Liang W, Gu X, Li X, et al. Mapping the epidemic changes and risks of hemorrhagic fever with renal syndrome in Shaanxi Province, China, 2005-2016. *Sci Rep*. 2018;8(1):749. | Used in HFRS ecological modeling | No missing data |
|  | Forest | Area of forest (m² per capita) | Xiao H, Lin X, Gao L, et al. Ecology and geography of hemorrhagic fever with renal syndrome in Changsha, China. *BMC Infect Dis*. 2013; 13:305. | Used in HFRS ENMs model | No missing data |
|  | Shrubland | Area of shrubland (m² per capita) | Riojas-López ME, Mellink E, Luévano J. A semiarid fruit agroecosystem as a conservation-friendly option for small mammals in an anthropized landscape in Mexico. *Ecol Appl*. 2018;28(2):495-507. | Used in ecological modeling of rodents | No missing data |
|  | Grassland | Area of grassland (m² per capita) | He J, Christakos G, Wu J, et al. Spatiotemporal variation of the association between climate dynamics and HFRS outbreaks in Eastern China during 2005-2016 and its geographic determinants. *PLoS Negl Trop Dis*. 2018;12(6): e0006554. | Used in HFRS ecological modeling | No missing data |
|  | Water body | Area of inland water body (m² per capita) | Shen L, Sun M, Wei X, et al. Spatiotemporal association of rapid urbanization and water-body distribution on hemorrhagic fever with renal syndrome: A case study in the city of Xi'an, China. *PLoS Negl Trop Dis*. 2022;16(1): e0010094. | Used in HFRS ecological modeling | No missing data |
|  | Snow | Area of snow (m² per capita) | Khalil H, Olsson G, Ecke F, et al. The importance of bank vole density and rainy winters in predicting nephropathia epidemica incidence in Northern Sweden. *PLoS One*. 2014;9(11): e111663. | Used in ecological modeling of bank vole | No missing data |
|  | Barren | Area of barren (m² per capita) | Tarasov MA, Garanina SB, Kresova UA, et al. Criteria of difference evaluation for-various types of hemorrhagic fever with renal syndrome foci. HFRS foci in various biotopes of northern barrens. *Zh Mikrobiol Epidemiol Immunobiol*. 2015;(2):74-80. | Used in HFRS ecological modeling | No missing data |
|  | Impervious surface | Area of impervious surface (m² per capita) | Xiao H, Lin X, Gao L, et al. Ecology and geography of hemorrhagic fever with renal syndrome in Changsha, China. *BMC Infect Dis*. 2013; 13:305. | Used in HFRS ENMs model | No missing data |
|  | Wetland | Area of wetland (m² per capita) | Hu WS, Liu W, Liu Y, et al. Epidemic characteristics and dynamic changes of spatio-temporal distribution of hemorrhagic fever with renal syndrome in Guangzhou, 2010-2019 *Zhonghua Liu Xing Bing Xue Za Zhi*. 2020;41(12):2087-2092. |  | No missing data |
| NDVI | Normalized difference vegetation index | Annual normalized difference vegetation index | He J, Wang Y, Mu D, et al. The Impacts of Climatic Factors and Vegetation on Hemorrhagic Fever with Renal Syndrome Transmission in China: A Study of 109 Counties. *Int J Environ Res Public Health*. 2019;16(18):3434. | Used in meta-analysis of HFRS | No missing data |
| Elevation | Elevation | Average elevation (m) | She K, Li C, Qi C, et al. Epidemiological Characteristics and Regional Risk Prediction of Hemorrhagic Fever with Renal Syndrome in Shandong Province, China. *Int J Environ Res Public Health.* 2021;18(16):8495. | Used in HFRS ecological modeling | No missing data |
| Urbanization-related factors | | | | |  |
| Land used for urban construction | Land used for urban construction | Area of land used for urban construction in districts under city (m² per capita). refer to the total area of all kinds of lands such as the residential land, the land for public administration and public service facilities, the land for commercial service facilities, industrial land, the land for logistics and warehouse, the land for road traffic facilities, the land for public facilities, green space and square land | Li J, Li W, Li B, et al. Construction Land Expansion of Resource-Based Cities in China: Spatiotemporal Characteristics and Driving Factors. *Int J Environ Res Public Health*. 2022;19(23):16109. | Represents an aspect of urbanization | Lack of data for 2020 |
| Park green land | Park green land | Area of parks green land in districts under city (m² per capita). refer to the total area occupied for green projects at the end of the reference  period, including park green land, production green land, protection green land, green land attached to institutions, and other green areas | Ofori BY, Garshong RA, Gbogbo F, Owusu EH, Attuquayefio DK. Urban green area provides refuge for native small mammal biodiversity in a rapidly expanding city in Ghana. *Environ Monit Assess*. 2018;190(8):480. | Urbanization (park green land) negatively influenced the abundance, diversity, and community composition of small mammals. | Lack of data for 2020 |
|  |  |  | Mexia T, Vieira J, Príncipe A, et al. Ecosystem services: Urban parks under a magnifying glass. *Environ Res*. 2018; 160:469-478. | Represents an aspect of urbanization | Lack of data for 2020 |
| Green land | Green land | Area of green land in districts under city (m² per capita). refer to the green area of various open parks | Blasdell KR, Morand S, Laurance SGW, et al. Rats and the city: Implications of urbanization on zoonotic disease risk in Southeast Asia. *Proc Natl Acad Sci U S A*. 2022;119(39): e2112341119. | Rodent species diversity decreased with increasing urbanization | Lack of data for 2020 |
|  |  |  | Qiu L, Pan Y, Zhu J, et al. Integrated analysis of urbanization-triggered land use change trajectory and implications for ecological land management: A case study in Fuyang, China. *Sci Total Environ*. 2019; 660:209-217. | Represents an aspect of urbanization |  |
| Built-up land | Built-up land | Area of built districts in districts under city (m² per capita). refer to the total area that were developed and constructed, with the basic municipal public facilities | Bordes F, Herbreteau V, Dupuy S, et al. The diversity of microparasites of rodents: a comparative analysis that helps in identifying rodent-borne rich habitats in Southeast Asia. *Infect Ecol Epidemiol*. 2013; 3:10.3402/iee.v3i0.20178. | Used in ecological modeling of rodents | Lack of data for 2020 |
|  |  |  | Chuai X, Huang X, Lu Q, et al. Spatiotemporal Changes of Built-Up Land Expansion and Carbon Emissions Caused by the Chinese Construction Industry. *Environ Sci Technol*. 2015;49(21):13021-13030. | Represents an aspect of urbanization |  |
| Real GDP | Real GDP | The inflation-adjusted measure that reflects the value of all goods and services produced by an economy in city (USD$ per capita) | Xiao H, Tian HY, Gao LD, et al. Animal reservoir, natural and socioeconomic variations and the transmission of hemorrhagic fever with renal syndrome in Chenzhou, China, 2006-2010. *PLoS Negl Trop Dis*. 2014;8(1): e2615. | Used in principal component analysis of HFRS | Lack of data for 2020 |
|  |  |  | Bai X, Chen J, Shi P. Landscape urbanization and economic growth in China: positive feedbacks and sustainability dilemmas. *Environ Sci Technol.* 2012;46(1):132-139. | Represents an aspect of urbanization |  |
| Population density | Population density | Average population per km² in city | Liang W, Gu X, Li X, et al. Mapping the epidemic changes and risks of hemorrhagic fever with renal syndrome in Shaanxi Province, China, 2005-2016. *Sci Rep.* 2018;8(1):749. | Used in HFRS ecological modeling | No missing data |
|  |  |  | Wu F, Wang X, Ren Y. Urbanization's Impacts on Ecosystem Health Dynamics in the Beijing-Tianjin-Hebei Region, China. *Int J Environ Res Public Health*. 2021;18(3):918. | Represents an aspect of urbanization |  |
| Nightlight index | Nightlight index | Average nightlight index in city | Bu L, Dai D, Tu L, et al. An STP-HSI index method for urban built-up area extraction based on multi-source remote sensing data. *R Soc Open Sci*. 2022;9(11):220597. | Represents an aspect of urbanization | No missing data |
| Electricity consumption | Electricity consumption | Total amount of electricity consumption in city (kWh) | Hua W, Wang L, Fang X, et al. Urbanization and energy equity: an urban-rural gap perspective. *Environ Sci Pollut Res Int*. 2023;30(16):46847-46868. | Represents an aspect of urbanization | No missing data |
| Real estate investment | Real estate investment | Investment actually completed by enterprises for real estate development (10,000 yuan per capita) | Chen Y, Lee CC. The impact of real estate investment on air quality: evidence from China. *Environ Sci Pollut Res Int*. 2020;27(18):22989-23001. | Represents an aspect of urbanization | Lack of data for 2020 |
| Floor space of commercialized buildings sold | Floor space of commercialized buildings sold | Floor space of commercialized buildings sold (m² per capita) | Zhang S, Li Z, Ning X, et al. Gauging the impacts of urbanization on CO2 emissions from the construction industry: Evidence from China. *J Environ Manage.* 2021;288:112440. | Represents an aspect of urbanization | Lack of data for 2020 |

* These datasets were extracted from the NASA Socioeconomic Data and Applications Center (SEDAC) Gridded Species Distribution collection created from vector data files acquired from the International Union for Conservation of Nature (IUCN) Red List collection. The data represent the species of mammals at one kilometer resolution. NDVI: normalized difference vegetation index. GDP: gross domestic product.

**Supplementary Table 3. Index of retrieved literatures for two hantavirus genotypes.**

| **Type of hantavirus** | **Number of studies (n)** | **Reference ID** |
| --- | --- | --- |
| HTNV | 96 | 1, 2, 3, 4, 5, 6, 7, 8, 9, 10, 11, 12, 13, 14, 15, 16, 17, 18, 19, 20, 21, 22, 23, 24, 25, 26, 27, 28, 29, 30, 31, 32, 33, 34, 35, 36, 37, 38, 39, 40, 41, 42, 43, 44, 45, 46, 47, 48, 49, 50, 51, 52, 53, 54, 55, 56, 57, 58, 59, 60, 61, 62, 63, 64, 65, 66, 67, 68, 69, 70, 71, 72, 73, 74, 75, 76, 77, 78, 79, 80, 81, 82, 83, 84, 85, 86, 87, 88, 89, 90, 91, 92, 93, 94, 95, 96 |
| SEOV | 112 | 1, 4, 6, 7, 8, 9, 10, 11, 15, 17, 18, 19, 22, 23, 24, 25, 29, 30, 34, 35, 37, 39, 40, 41, 42, 44, 45, 46, 51, 52, 53, 55, 56, 57, 58, 59, 60, 69, 84, 86, 95, 97, 98, 99, 100, 101, 102, 103, 104, 105, 106, 107, 108, 109, 110, 111, 112, 113, 114, 115, 116, 117, 118, 119, 120, 121, 122, 123, 124, 125, 126, 127, 128, 129, 130, 131, 132, 133, 134, 135, 136, 137, 138, 139, 140, 141, 142, 143, 144, 145, 146, 147, 148, 149, 150, 151, 152, 153, 154, 155, 156, 157, 158, 159, 160, 161, 162, 163, 164, 165, 166, 167 |

**Supplementary Table 4. The annual trend change of urbanization-related factors of HTNV-dominant and SEOV-dominant cities during different phases.**

| Urbanization-related factors |  | 2001~2010 | | | | | | |  | 2011~2020 | | | | | | | | |
| --- | --- | --- | --- | --- | --- | --- | --- | --- | --- | --- | --- | --- | --- | --- | --- | --- | --- | --- |
|  |  | No. of HTNV-dominant cities | | |  | No. of SEOV-dominant cities | | |  | No. of HTNV-dominant cities | | | |  | | No. of SEOV-dominant cities | | |
|  |  | Decrease (%) | Fluctuation (%) | Increase (%) |  | Decrease (%) | Fluctuation (%) | Increase  (%) |  | Decrease (%) | Fluctuation (%) | Increase (%) |  | | Decrease (%) | | Fluctuation (%) | Increase (%) |
| Population density |  | 5 (33.33) | 1 (6.67) | 9 (60.00) |  | 42 (24.85) | 3 (1.78) | 124 (73.37) |  | 7 (53.85) | 2 (15.38) | 4 (30.77) |  | | 34 (22.22) | | 0 (0) | 119 (77.78) |
| Real GDP |  | 0 (0) | 0 (0) | 15 (100) |  | 0 (0) | 5 (2.96) | 164 (97.04) |  | 0 (0) | 0 (0) | 13 (100) |  | | 0 (0) | | 0 (0) | 153 (100) |
| Nightlight index |  | 0 (0) | 1 (6.67) | 14 (93.33) |  | 0 (0) | 5 (2.96) | 164 (97.04) |  | 0 (0) | 8 (61.54) | 5 (38.46) |  | | 0 (0) | | 35 (22.88) | 118 (77.12) |
| Land used for urban construction |  | 1 (6.67) | 9 (60.00) | 5 (33.33) |  | 11 (6.51) | 64 (37.87) | 94 (55.62) |  | 1 (7.69) | 6 (46.15) | 6 (46.15) |  | | 20 (13.07) | | 91 (59.48) | 42 (27.45) |
| Park green land |  | 1 (6.67) | 5 (33.33) | 9 (60.00) |  | 4 (2.37) | 47 (27.81) | 118 (69.82) |  | 0 (0) | 6 (46.15) | 7 (53.85) |  | | 8 (5.23) | | 68 (44.44) | 77 (50.33) |
| Green land |  | 2 (13.33) | 3 (20.00) | 10 (66.67) |  | 9 (5.33) | 46 (27.22) | 114 (67.46) |  | 1 (7.69) | 3 (23.08) | 9 (69.23) |  | | 10 (6.54) | | 68 (44.44) | 75 (49.02) |
| Built-up land |  | 2 (13.33) | 2 (13.33) | 11 (73.33) |  | 3 (1.78) | 55 (32.54) | 111 (65.68) |  | 0 (0) | 5 (38.46) | 8 (61.54) |  | | 16 (10.46) | | 86 (56.21) | 51 (33.33) |
| Electricity consumption |  | 0 (0) | 0 (0) | 15 (100) |  | 0 (0) | 5 (2.96) | 164 (97.04) |  | 0 (0) | 0 (0) | 13 (100) |  | | 0 (0) | | 0 (0) | 153 (100) |
| Real estate investment |  | 0 (0) | 0 (0) | 15 (100) |  | 0 (0) | 5 (2.96) | 164 (97.04) |  | 0 (0) | 8 (61.54) | 5 (38.46) |  | | 7 (4.58) | | 30 (19.61) | 116 (75.82) |
| Floor space of commercialized buildings sold | | 0 (0) | 2 (13.33) | 13 (86.67) |  | 1 (0.59) | 13 (7.69) | 155 (91.72) |  | 3 (23.08) | 4 (30.77) | 6 (46.15) |  | | 10 (6.54) | | 37 (24.18) | 106 (69.28) |
| Cropland |  | 4 (26.67) | 11 (73.33) | 0 (0) |  | 129 (76.33) | 34 (20.12) | 6 (3.55) |  | 3 (23.08) | 9 (69.23) | 1 (7.69) |  | | 92 (60.13) | | 39 (25.49) | 22 (14.38) |
| Forest |  | 4 (26.67) | 9 (60.00) | 2 (13.33) |  | 58 (34.32) | 83 (49.11) | 27 (15.98) |  | 6 (46.15) | 4 (30.77) | 3 (23.08) |  | | 80 (52.29) | | 44 (28.76) | 29 (18.95) |
| Impervious surface |  | 0 (0) | 0 (0) | 15 (100) |  | 0 (0) | 11 (6.51) | 158 (93.49) |  | 0 (0) | 0 (0) | 13 (100) |  | | 0 (0) | | 1 (0.65) | 152 (99.35) |

The trend test was calculated by the Mann-Kendall test.

**Supplementary Figure 1. The flow diagram of literature review.**

**
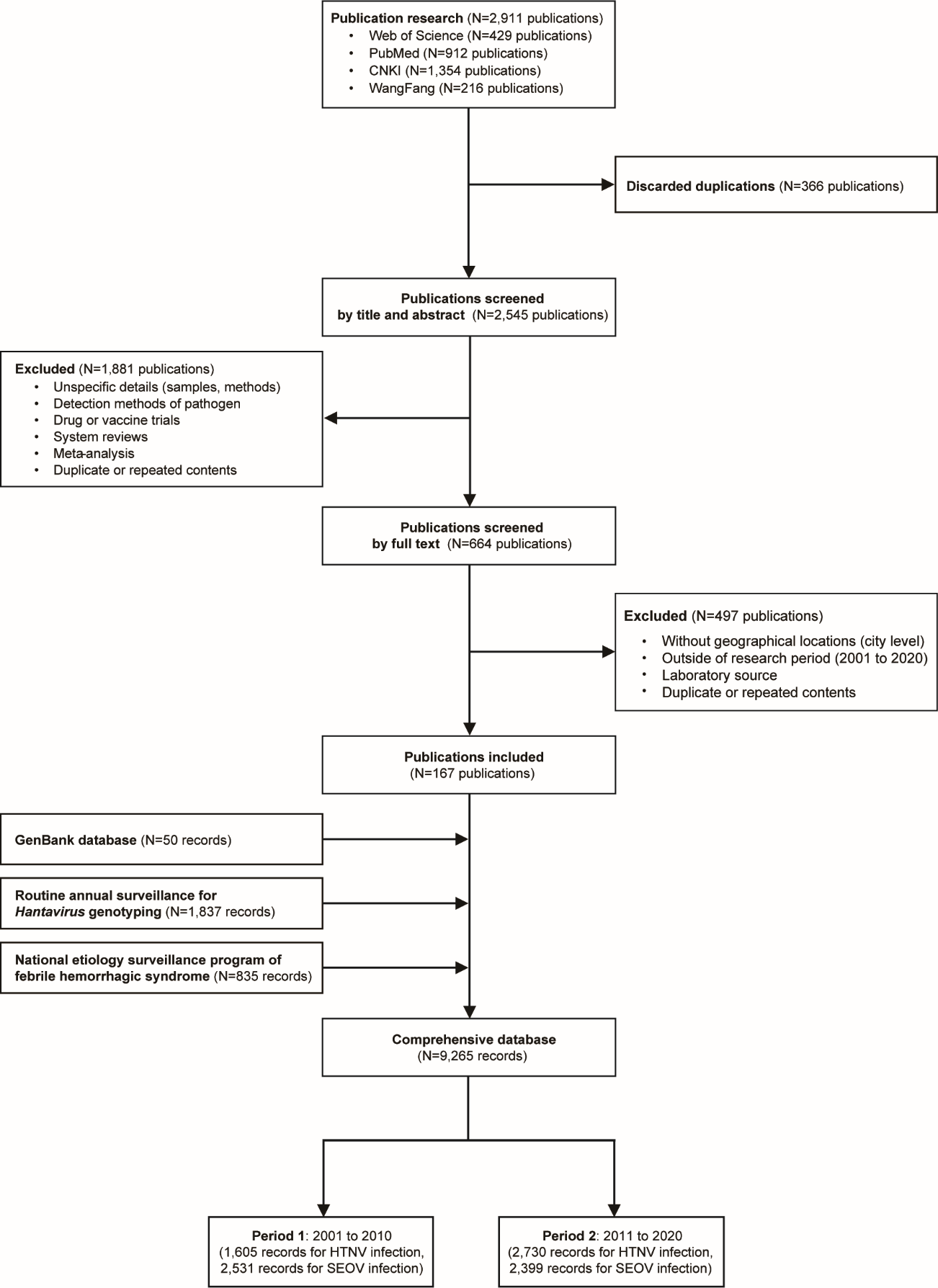
**

**Supplementary Figure 2. The observed geographical distributions of** **HTNV and SEOV during two ten-year intervals.** The grey background indicates the areas with no genotyping data or no risk, and the other two colored backgrounds indicate the observed areas of HTNV and SEOV. HTNV: Hantaan virus. SEOV: Seoul virus.

**
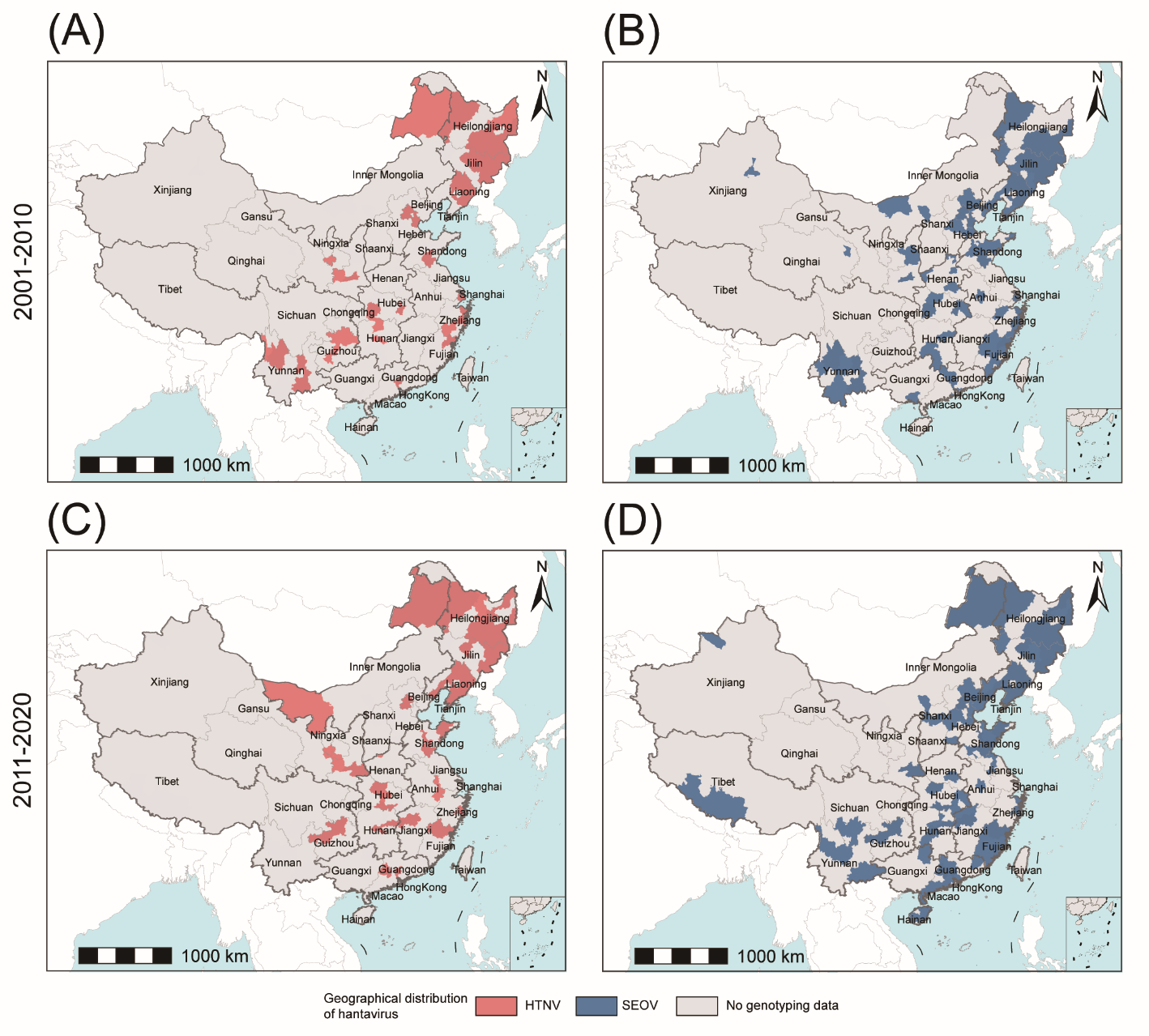
**

**Supplementary Figure 3.** **Effects of major variables (top nine) for predicting the proportion of HTNV based on the two-stage XGBoost model from 2001 to 2010.** The median curves with interquartile range show the influence of those variables on predicting the proportion of HTNV. The histograms show the frequency distributions of the predictors. XGBoost: extreme gradient boosting HTNV: Hantaan virus. SEOV: Seoul virus. GDP: gross domestic product.


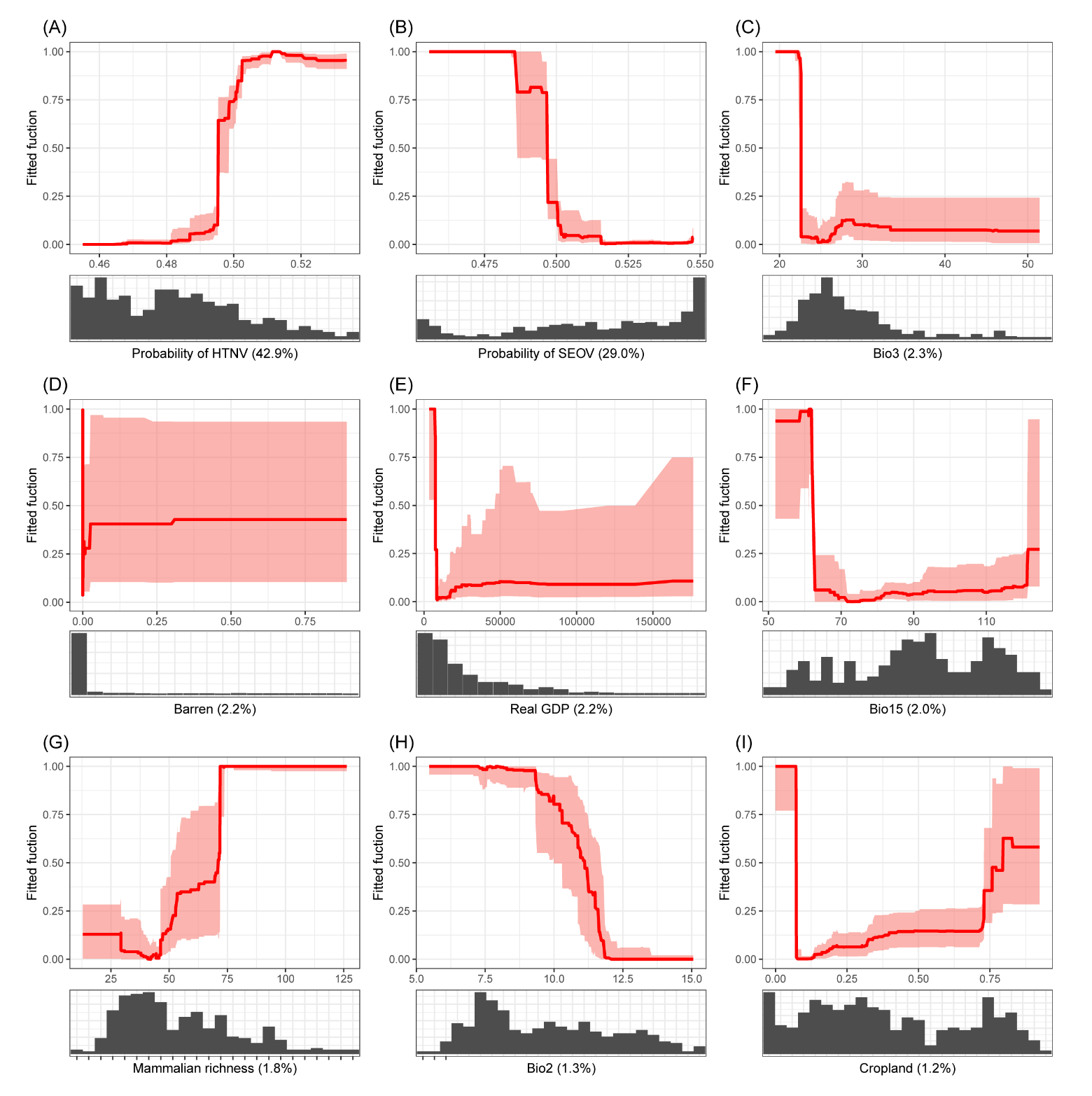


**Supplementary Figure 4.** **Effects of major variables (top nine) for predicting the proportion of HTNV based on the two-stage XGBoost model from 2011 to 2020.** The median curves with interquartile range show the influence of those variables on predicting the proportion of HTNV. The histograms show the frequency distributions of the predictors. XGBoost: extreme gradient boosting HTNV: Hantaan virus. SEOV: Seoul virus.


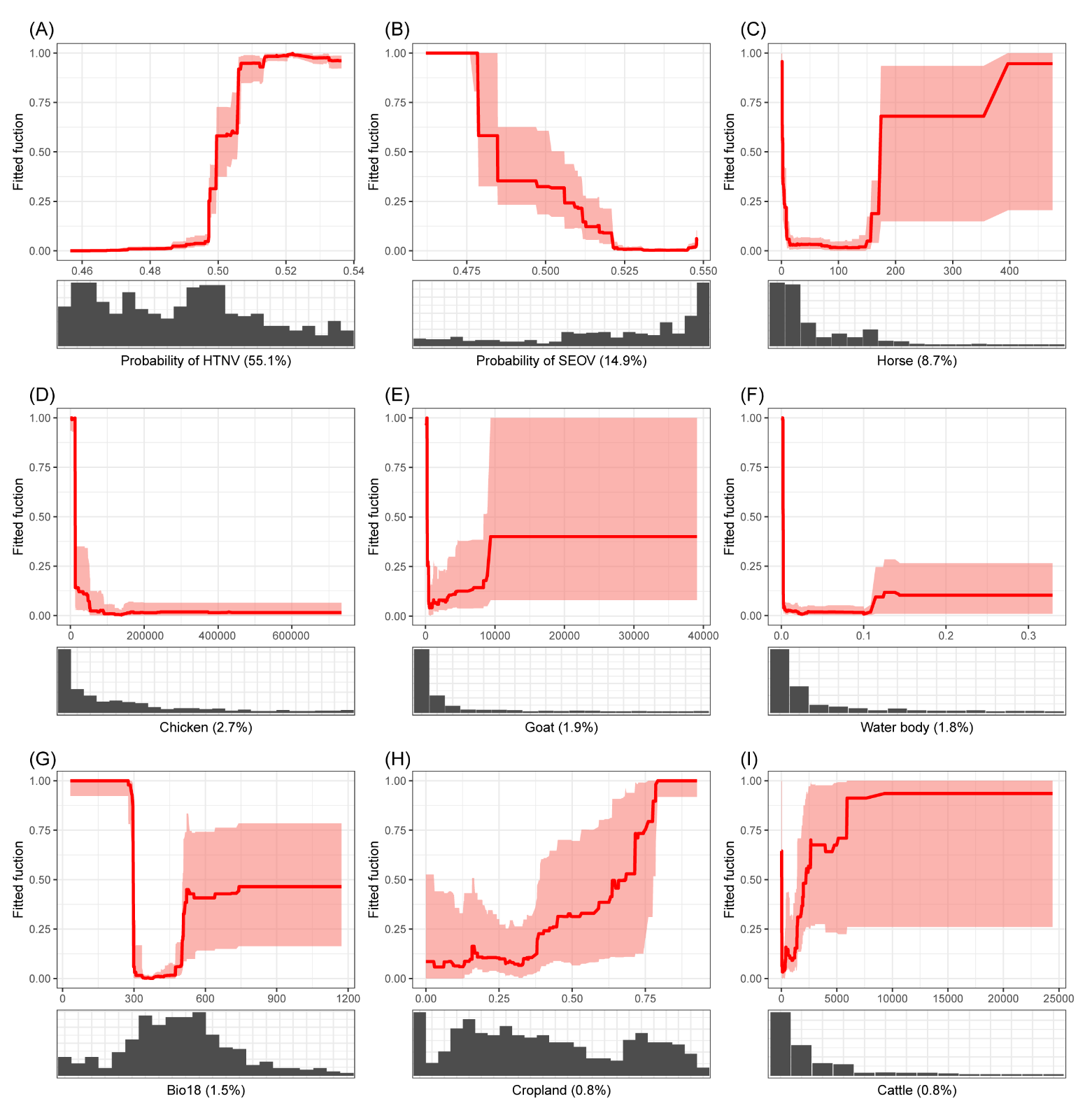


**Supplementary Figure 5. Effects of** **major** **variables (top nine)** **indicated by SHAP values in the optimal modelling (XGBoost) for patients infected with HTNV and SEOV.** (A) Projected proportion of HTNV. (B) White blood cell count. (C) Bellyache. (D) Vomiting. (E) Pantalgia. (F) Sex. (G) Age. (H) Platelet count. (I) Conjunctival congestion. The x-axis indicates the observed values of major variables, while the y-axis indicates the SHAP values. In the plots of continuous variables (A, B, G and H), the scattered points indicate the distribution of fitting results for each city every year, and the red lines represent the pooled exposure-response curves with their 95% CI indicated by the shaded areas. Box plots are used for the SHAP values of the categorical variables (C, D, E, F and I). The absence (no) or presence (yes) of a symptom is indicated by a blue or red colored box, respectively. SHAP: shapley additive explanations. XGBoost: extreme gradient boosting. HTNV: Hantaan virus. CI: confidence interval.


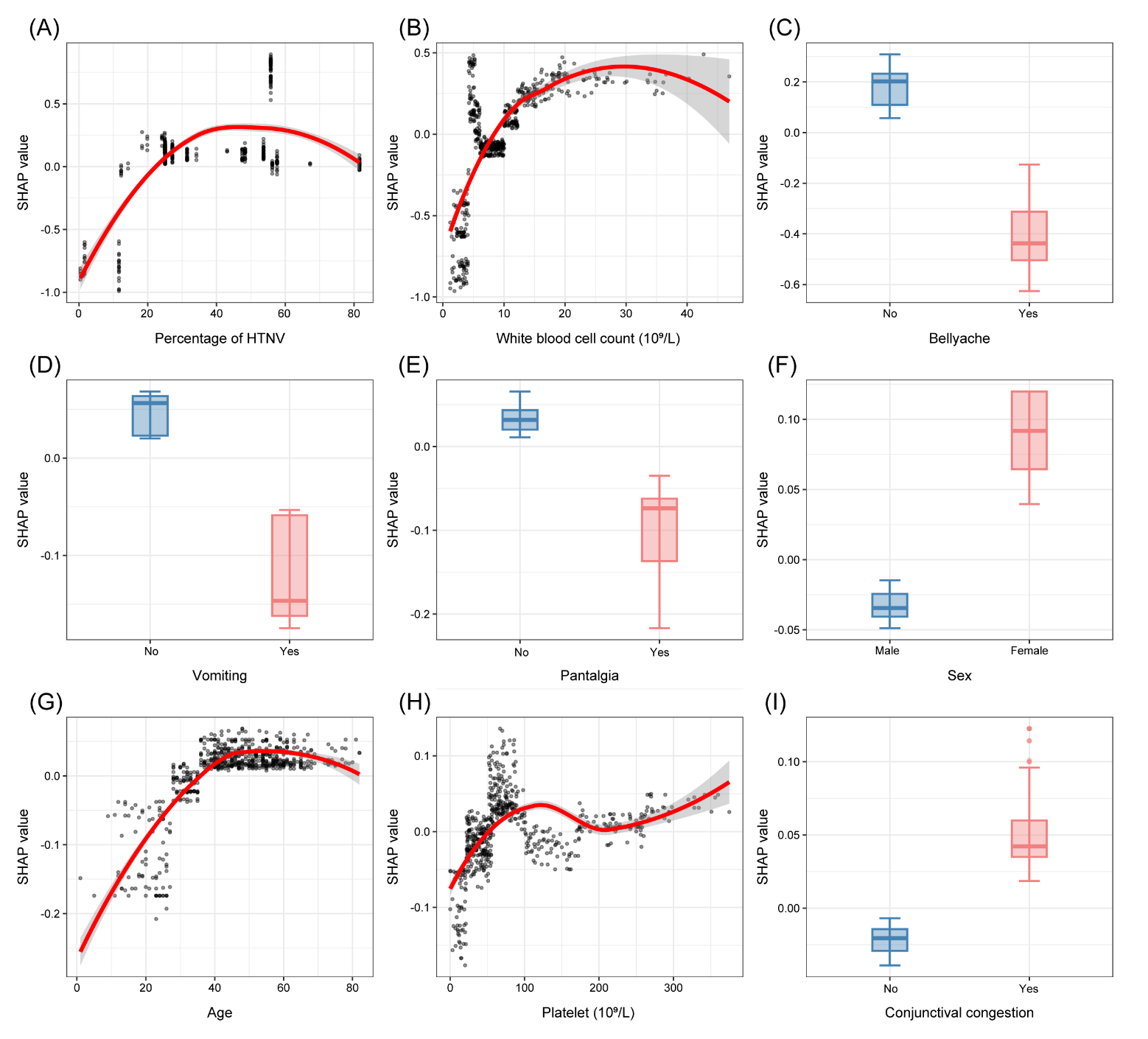


**Supplementary Figure 6. Comparison of modelling for the association of urbanization-related factors with HFRS incidence.** (A) Modelling for HTNV-dominant cities. (B) Modelling for SEOV-dominant cities. RMSE, MAE, NRMSE and RMSLE were processed as the reciprocal of original values for plotting. HTNV: Hantaan virus. SEOV: Seoul virus. RMSE: root mean squared error. MAE: mean absolute error. NRMSE: normalized root mean squared error. RMSLE: root mean squared logarithmic error. XGBoost: extreme gradient boosting. RF: random forest. GBM: gradient boosting machine. GLM: generalized linear model.


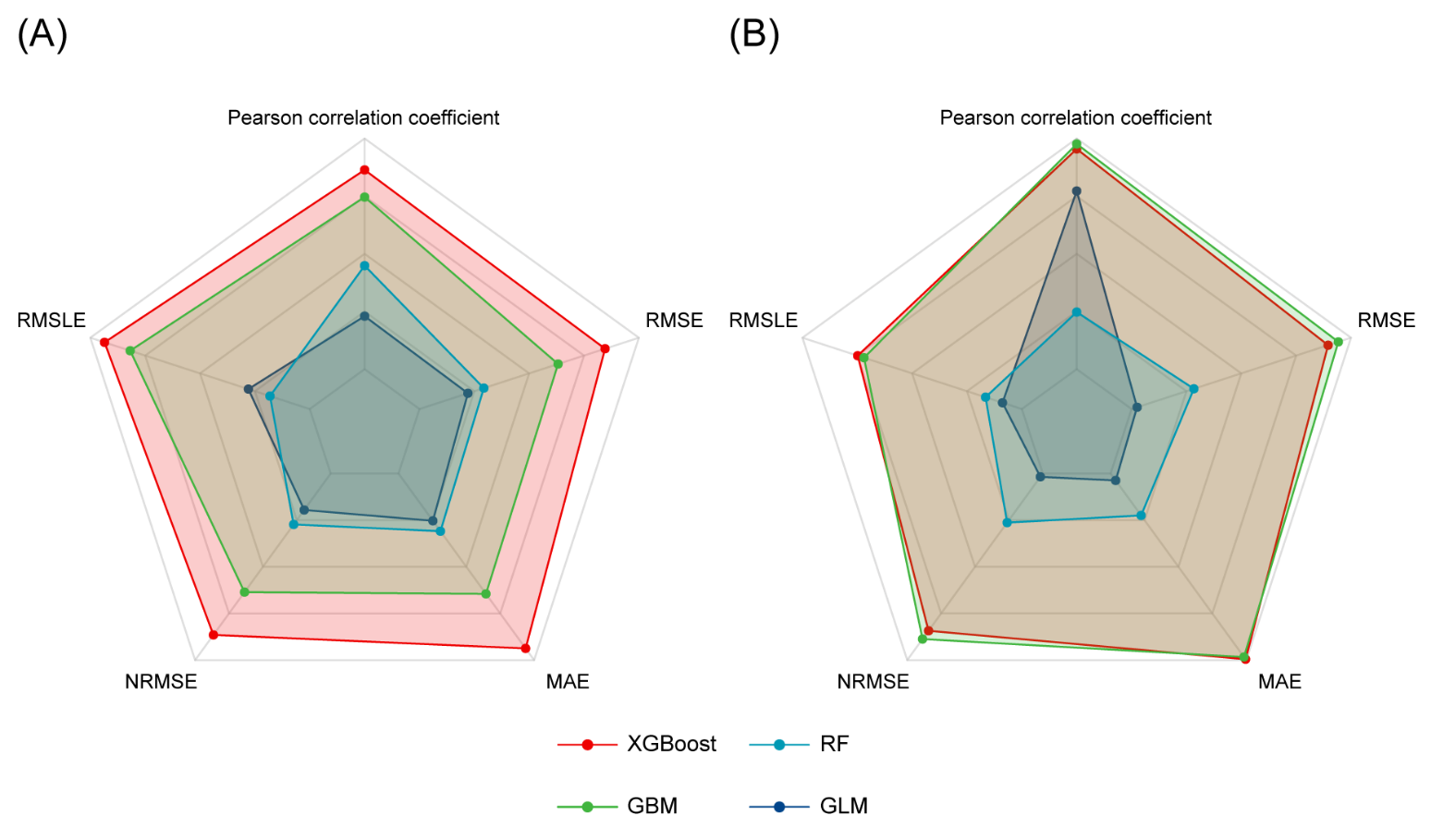


**Supplementary Figure 7.** **Importance and effects of variables based on the XGBoost model for the association of urbanization-related factors with HFRS incidence.** (A) Summary of SHAP values in modelling for HTNV-dominant cities. (B) Summary of SHAP values in modelling for SEOV-dominant cities. The variables are ranked in the importance according to their global SHAP values from top to bottom, with the y-axis indicating different variables and the x-axis indicating the SHAP values. Colors from yellow to purple indicate feature values from low to high. SHAP: shapley additive explanations. XGBoost: extreme gradient boosting. HTNV: Hantaan virus. SEOV: Seoul virus. GDP: gross domestic product. LUFUC: land used for urban construction. FSOCBS: floor space of commercialized buildings sold.

**
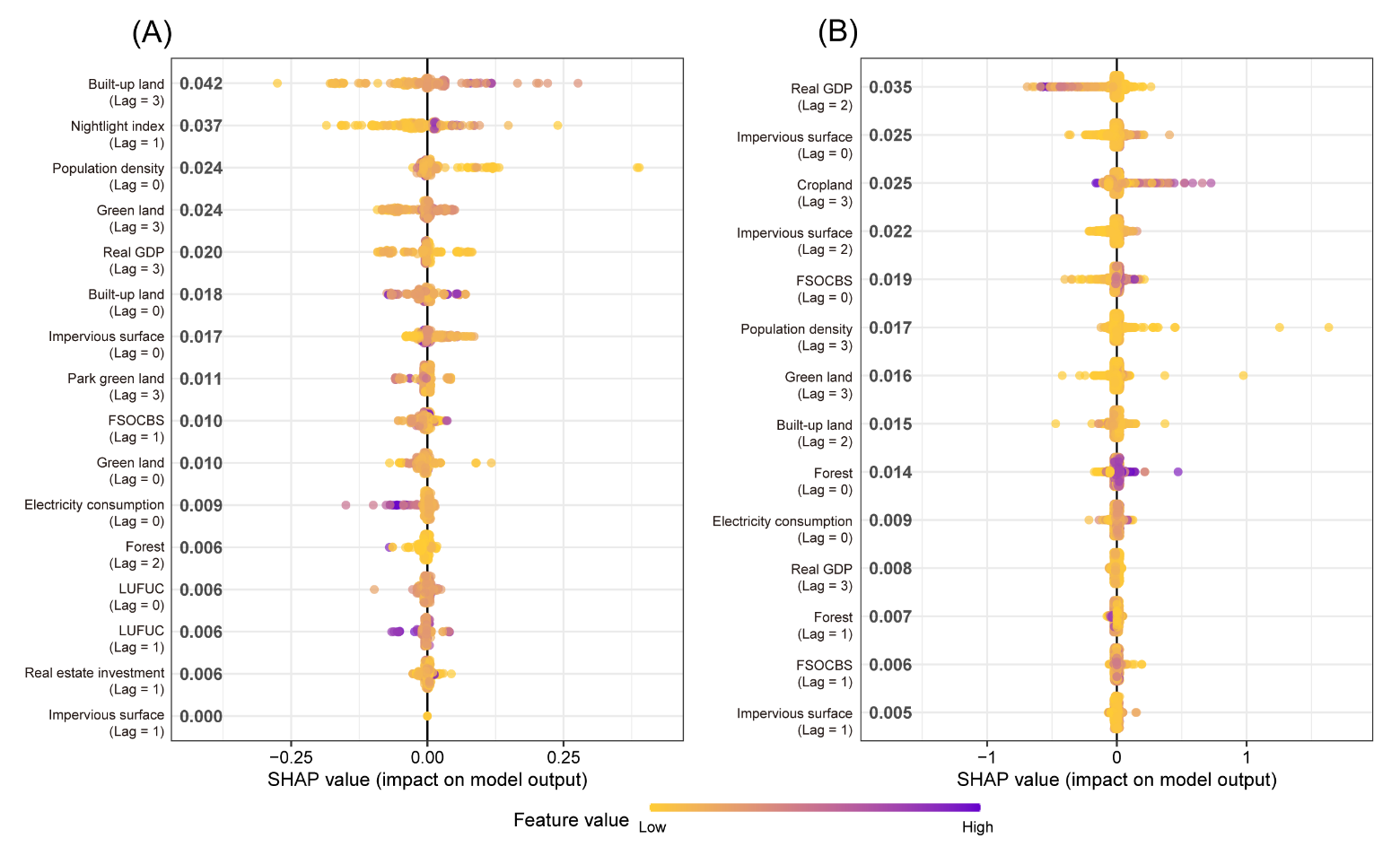
**

**Reference 2**

1. Shang C. Molecular epidemiologic characterization of hantaviruses in China [Degree of master]: Chinese Center for Disease Control and Prevention; 2021.

2. Li X., Dong X., Zhao C. Z., et al. Genetic isolation and identification of hantavirus. *Chinese Preventive Medicine* 2004; (06): 30-2.

3. Jin T. Z., Ma C. F., Zheng H. C., et al. Hantaviruses in the Lesser White-toothed Shrew (Crocidura suaveolens) in Xi'an. *Journal of Pathogen Biology* 2011; 6(08): 565-6+56.

4. Xu F., Zhu H. P., Yang Z. N., et al. The host investigation and virus isolation of hantavirus in Tiantai County. *Preventive Medicine* 2019; 31(05): 433-6+40.

5. Dong X., Li X., Zhao C. Z., Wang B. Isolation and phylogenetic analysis of one strain of hantavirus in Shenyang. *Chinese Journal of Health Laboratory Technology* 2007; (10): 1763-5.

6. Li Y. T., Zhou Y., Zhou X., Shen R. M., Shen W. J. The classification and sequence analysis of hantavirus genes in Shanghai. *Chinese Journal of Viral Diseases* 2004; (04): 216-8.

7. Zhuo M. Y., Zhang Z. P., Cai C. H., Wen H., Jiang X. M. Epidemiological characteristics of epidemic hemorrhagic fever in Nanping City, 2016-2019. *Strait Journal of Preventive Medicine* 2021; 27(03): 26-8.

8. Yao L. S., Huang B., Shi D. W., Cheng Y. S., Bai G. L. Investigation of the source of renal syndrome hemorrhagic fever in Panshi City, Jilin Province, China. *Chinese Journal of Disease Control and Prevention* 2004; (02): 191.

9. Li Y., Zhang Y. Z., Xiao Q. Y., Minghui L., Huaxin C. Epidemiologic study of hantaviruses in rodents in Hunan Province, China. National Symposium on the Prevention and Control of Zoonoses; 2006; Beijing, China; 2006. p. 2.

10. Dai D. F., Zhang H., Liu Y. Z., et al. Virological surveillance on hemorrhagic fever with renal sydrome in Hunan province in 2006. *Chinese Journal of Epidemiology* 2007; 28(12): 1194-7.

11. Liu T., Huang J. G., Hao H. B., et al. Epidemic situation of hemorrhagic fever with renal syndrome and investigation of the host animals in Jingzhou city of Hubei province in 2017. *Chinese Journal of Vector Biology and Control* 2018; 29(06): 65-8.

12. Wang C. Q. The molecular epidemiology investigation of HV on the Bolshoy Ussuriysky Island: North China University of Science and Technology; 2015.

13. Sun Y. F., Hu K., Wu Y. P., Tian H. Study on genotype and gene sequence characters of Hantavirus in Baoji. *Occupation and Health* 2016; 32(08): 1049-52.

14. Liu S. W., Gong T. Y., Xu G., et al. Complete genome sequence analysis of Hantaan virus strain AYW89-15isolated in Jiangxi, China. *Chinese Journal of Zoonoses* 2017; 33(12): 1089-93.

15. Zheng Z., Wang P., Wang Z., et al. The characteristics of current natural foci of hemorrhagic fever with renal syndrome in Shandong Province, China, 2012-2015. *PLoS Negl Trop Dis* 2019; 13(5): e0007148.

16. Li H. B., Fu H. L., Hu K., et al. Survey on host animals of hemorrhagic fever with renal syndrome in Baoji City of Shaanxi Province from 2014 to 2018. *Chinese Journal of Hygienic Insecticides & Equipments* 2020; 26(06): 529-32.

17. Wang Y. L., Cai D., Xu Y., Jin Z., Rukun F., Shulu S. Surveillance on rats and hantavirus detection in Rizhao plain area. *Chinese Journal of Frontier Health and Quarantine* 2016; 39(06): 417-9+06.

18. Jing Q. L., Luo L., Xiao X. C., et al. Surveillance on hemorrhagic fever with renal syndrome in Guangzhou, 2001-2010. *Journal of Tropical Medicine* 2012; 12(01): 15-8+33.

19. Chen Y., Lin D. H., Chen L., et al. Surveillance of epidemic and host animals of hemorrhagic fever with renal syndrome in 2013-2015 in Fujian province, China. *Chinese Journal of Vector Biology and Control* 2016; 27(06): 594-6.

20. Wang W., Wang M. R., Lin X. D., et al. Ongoing spillover of Hantaan and Gou hantaviruses from rodents is associated with hemorrhagic fever with renal syndrome (HFRS) in China. *PLoS Negl Trop Dis* 2013; 7(10): e2484.

21. Tian H., Tie W. F., Li H., et al. Orthohantaviruses infections in humans and rodents in Baoji, China. *PLoS Negl Trop Dis* 2020; 14(10): e0008778.

22. Fang L. Z., Zhao L., Wen H. L., et al. Reservoir host expansion of hantavirus, China. *Emerg Infect Dis* 2015; 21(1): 170-1.

23. Yao L. S. Preliminary study on rodents, body surface parasites and their pathogens in the port area adjacent to Changbai Mountain of China and Korea; 2012.

24. Lin C., Guo J., Xu Q. W. Monitoring results of hemorrhagic fever with renal syndrome in Zhouning county from 2011 to 2015. *Strait Journal of Preventive Medicine* 2016; 22(05): 23-5.

25. Zhang P., Chen Y. W., Duan Y. B., Tao H., Yanliang Q., Qiang L. Monitoring and analysis of host animals of hemorrhagic fever with renal syndrome in Shangluo City, Shaanxi Province from 2013 to 2015. *Journal of Medical Pest Control* 2020; 36(01): 63-5+8.

26. Shen B., Piao D. F., Wu D. L., et al. Isolation and identification of hantavirus in Fusong area. *Chinese Journal of Public Health Engineering* 2012; 11(02): 97-9+103.

27. Xu F., Zhu H. P., Yao P. P., et al. Isolation and identification of Hantavirus carried by rodents in Zhejiang province, China from 2008 to 2011. *Chinese Journal of Vector Biology and Control* 2013; 24(04): 285-8.

28. Wang M. R., Wang W., Lin X. D., Mei S. H., Guo W. P., Zhang Y. Z. Investigation on the natural infectious status of hantaviruses among small mammals in Longquan city, Zhejiang province. *Chinese Journal of Epidemiology* 2011; 32(6): 598-601.

29. Chen W. Q., Luo W. L., Huang J. Y., Li B. Q. Survey on Hantavirus infection of rat and health people in Qingyuan City. *Occupation and Health* 2018; 34(22): 3149-51.

30. Cui B. J., Yi H. H., Wu B. Y., et al. Investigation and analysis on pathogens carried by rodents at ports of Jiangsu from 2014 to 2015. *Chinese Journal of Hygienic Insecticides & Equipments* 2016; 22(01): 73-6.

31. Lu T., Fu Y., Hou Y., et al. Hantavirus RNA Prevalence in Myomorph Rodents on Bolshoy Ussuriysky Island at the Sino-Russian Border. *Vector Borne Zoonotic Dis* 2017; 17(8): 588-95.

32. Ma C., Yu P., Nawaz M., et al. Hantaviruses in rodents and humans, Xi'an, PR China. *J Gen Virol* 2012; 93(Pt 10): 2227-36.

33. Ma X. M., Ma J. T., Chen H., Yi Y., Zheng Z. Hantavirus was isolated from the lungs of social rodents for the first time in Ningxia. *Chinese Journal of Preventive Medicine* 2012; 46(4): 373-5.

34. Cao Z. W. Infection and genetic variation of hantavirus in small mammals in some region of China [Degree of master]: Academy of Military Sciences of PLA; 2010.

35. Wang A. N., Dang Y. Q., Li B. Q., Yongzhong L., Zhu L. Genotyping and sequence analysis of wild animals carring viruses in Raohe area, China. *Chinese Journal of Zoonoses* 2020; 36(03): 206-10.

36. Hu Q., Tong S. M., Guo L. P. Genotyping and DNA Sequenece Analysis of Hantaviruses at Daxie Port. *Chinese Journal of Frontier Health and Quarantine* 2010; 33(04): 224-8.

37. Yao P. P., Chen G., Xu F., et al. Genotype and evolutionary variation of hantavirus in Tiantai County, Zhejiang Province from 2011 to 2018. *Chinese Journal of Epidemiology* 2019; 40(10): 1285-90.

38. Geng Y. Z., Tian J., Liu Y., et al. Genetic features and distribution of Hantaan virus in Liaoning province, China. *Chinese Journal of Vector Biology and Control* 2012; 23(05): 449-51+54.

39. Li J. L., Ling J. X., Liu D. Y., et al. Genetic characterization of a new subtype of Hantaan virus isolated from a hemorrhagic fever with renal syndrome (HFRS) epidemic area in Hubei Province, China. *Arch Virol* 2012; 157(10): 1981-7.

40. Cao S., Ma J., Cheng C., Ju W., Wang Y. Genetic characterization of hantaviruses isolated from rodents in the port cities of Heilongjiang, China, in 2014. *BMC Vet Res* 2016; 12: 69.

41. Liu J., Liu D. Y., Chen W., et al. Genetic analysis of hantaviruses and their rodent hosts in central-south China. *Virus Res* 2012; 163(2): 439-47.

42. Liu D. Y., Liu J., Li J. L., et al. enetic analysis on S segment of bantaviruses in rodent hosts from Wuhan area, Hubei province. *Chinese Journal of Epidemiology* 2019; 33(8): 828-31.

43. Lv T., Qiu D. Z., Zhang W. S. Epidemiological investigation and analysis of hemorrhagic fever with renal syndrome in Xiangyang City from 2009 to 2013. *Chinese Primary Health Care* 2015; 29(05): 91-2.

44. Li W. J., Zhang H. L., Zhang Y. Z., et al. Epidemiological characteristics of hemorrhagic fever with renal syndromein in Xiangyun County, Yunnan Province. *China Tropical Medicine* 2013; 13(11): 1319-22.

45. Bai J. J., Wang X. N., Li L., et al. Analysis on epidemiological characteristics of hemorrhagic fever with renal syndrome in Hubei, China during 2001-2015. *Modern Preventive Medicine* 2016; 43(20): 3655-60.

46. Liu J., Chen Y., Lin D. H., et al. Epidemiological characteristics and host animal monitoring of hemorrhagic fever with renal syndrome in Fujian province, China, 2016-2018. *Chinese Journal of Vector Biology and Control* 2021; 32(03): 329-33.

47. Wang C. Q., Gao J. H., Li M., et al. Co-circulation of Hantaan, Kenkeme, and Khabarovsk Hantaviruses in Bolshoy Ussuriysky Island, China. *Virus Res* 2014; 191: 51-8.

48. Wu Z., Du J., Lu L., et al. Detection of Hantaviruses and Arenaviruzses in three-toed jerboas from the Inner Mongolia Autonomous Region, China. *Emerg Microbes Infect* 2018; 7(1): 35.

49. Wu R., Yu P. B., Li J., et al. Complete genome sequencing and analysis of hantavirus Xi'an isolate. *Shaanxi Medical Journal* 2013; 42(12): 1581-2+5.

50. Yan Q. L., Yang P. F., Shao L. J., et al. Characteristics of S gene of hantavirus carried by Apodemus agrarius in Changbai County. *Chinese Journal of Virology* 2013; 29(04): 382-5.

51. Liu S. W., Xu G., Gong T., et al. Analysis on genetic characteristics of Hantavirus from rodents in Jiangxi province. *Chinese Journal of Vector Biology and Control* 2015; 26(05): 475-9.

52. Wang L., Liu F. Y., Jiang X. L., et al. Analysis on epidemiological characteristics of hemorrhagic fever with renal syndrome in Zibo city from 2006 to 2019. *Modern Preventive Medicine* 2021; 48(10): 1747-52.

53. He S., Chen Y., Lin W., Jiaxiong W., Shuyang L., Yanqin D. Analysis of monitoring results of hemorrhagic fever with renal syndrome in Fujian Province in 2010. *Strait Journal of Preventive Medicine* 2012; 18(03): 26-7.

54. Ma X. M., Ma J. T., Li H. J., Yi Y., Jun Z., Zheng Z. Analysis of host animal surveillance of renal syndrome hemorrhagic fever in Jingyuan County, Ningxia, 2005-2012. *Journal of Ningxia Medical University* 2014; 36(02): 187-90.

55. Liu T., Hao H. B., Yao M. L., et al. Analysis of hemorrhagic fever with syndrome host animal surveillance in Jingzhou City , Hubei Province from 2017 to 2020. *Chinese Journal of Zoonoses* 2022; 38(02): 128-34.

56. Zhao W. S., Tan H. Q., Cheng J. P., Xueen L., Lebin S., Qiang H. Surveillance and analysis on plague and other rodent-borne infectious diseases in Zhaoqing in 2019. *Journal of Medical Pest Control* 2022; 38(02): 132-5.

57. Wang L., Zhang Y., Zhang L., et al. Analysis of epidemiological characteristics and surveillance result of host animal on hemorrhagic fever with renal syndrome in Zibo City from 2015 to 2018. *Chinese Journal of Disease Control and Prevention* 2020; 24(02): 237-40.

58. Liu S. W., Xu G., Gong T., et al. Genetic types and sub-types analysis of orthohantavirus in rodents in Jiangxi Province from 2012 to 2015. *Modern Preventive Medicine* 2018; 45(06): 1090-4+113.

59. Li J., Liu T., Huang J. G., et al. Epidemiological characteristics and host animal investigation of hemorrhagic fever with renal syndrome in Jiangling County, Jingzhou City, 2010-2018. *Journal of Public Health and Preventive Medicine* 2019; 30(05): 45-9.

60. Zhang C. H., Xu Q. W., Liu J. R. Surveillance and Analysis of Hemorrhagic Fever with Renal Syndrome in Zhouning County, 2008-2009. *Preventive Medicine Tribune* 2011; 17(03): 193-5.

61. Chen X. Y., Lei Y. L., Mei S. H., et al. Surveillance of hemorrhagic fever with renal syndrome and virus isolation/identification in Longquan, 2005-2010. *Disease Surveillance* 2011; 26(11): 870-2.

62. Ma C., Wang Z., Li S., et al. Analysis of an outbreak of hemorrhagic fever with renal syndrome in college students in Xi'an, China. *Viruses* 2014; 6(2): 507-15.

63. Liao J. H., Zeng M. C., Zhou Y. P., Ouyang W., Wu Z. B. Epidemiologic characteristics and control effect of hemorrhagic fever with renal sgndrome in Zixing from 1991 to 2002. *Practical Preventive Medicine* 2004; (5): 922-3.

64. Wang M. L., Zhu Y., Wang J. P., Jingmei L., Liang F., Boquan J. Identification of HTNV-NP-specific T lymphocyte epitopes and analysis of epitope-specific T cell response. *Chinese Journal of Cellular and Molecular Immunology* 2005; (06): 45-7.

65. Ma Y., Liu B., Yuan B., et al. Sustained high level of serum VEGF at convalescent stage contributes to the renal recovery after HTNV infection in patients with hemorrhagic fever with renal syndrome. *Clin Dev Immunol* 2012; 2012: 812386.

66. Zhang Y., Wang M., Zhang X., et al. HTNV infection induces activation and deficiency of CD8+MAIT cells in HFRS patients. *Clin Exp Immunol* 2023; 211(1): 1-14.

67. Tian Z., Yao N., Wu Y., Wang F., Zhao Y. Serum superoxide dismutase level is a potential biomarker of disease prognosis in patients with hemorrhagic fever with renal syndrome caused by the Hantaan virus. *BMC Infect Dis* 2022; 22(1): 446.

68. Du H., Hu H., Wang P., et al. Predictive value of pentraxin-3 on disease severity and mortality risk in patients with hemorrhagic fever with renal syndrome. *BMC Infect Dis* 2021; 21(1): 445.

69. Liu S. W., Li J. X., Zou L., et al. Orthohantavirus infections in humans and rodents in the Yichun region, China, from 2016 to 2021. *PLoS Negl Trop Dis* 2023; 17(8): e0011540.

70. Zheng X. Y., Bian P. Y., Ye C. T., et al. Interferon-Induced Transmembrane Protein 3 Inhibits Hantaan Virus Infection, and Its Single Nucleotide Polymorphism rs12252 Influences the Severity of Hemorrhagic Fever with Renal Syndrome. *Front Immunol* 2016; 7: 535.

71. Tang K., Cheng L., Zhang C., et al. Novel Identified HLA-A*0201-Restricted Hantaan Virus Glycoprotein Cytotoxic T-Cell Epitopes Could Effectively Induce Protective Responses in HLA-A2.1/K(b) Transgenic Mice May Associate with the Severity of Hemorrhagic Fever with Renal Syndrome. *Front Immunol* 2017; 8: 1797.

72. Zhang Y., Zhang C., Zhuang R., et al. IL-33/ST2 correlates with severity of haemorrhagic fever with renal syndrome and regulates the inflammatory response in Hantaan virus-infected endothelial cells. *PLoS Negl Trop Dis* 2015; 9(2): e0003514.

73. Han D., Liu Z., Han Q., et al. Acute kidney injury in patients with hemorrhagic fever with renal syndrome caused by Hantaan virus: comparative evaluation by RIFLE and AKIN criteria. *Vector Borne Zoonotic Dis* 2011; 11(6): 723-30.

74. Liu J. M., Zhu Y., Xu Z. W., et al. Dynamic changes of apoptosis-inducing ligands and Th1/Th2 like subpopulations in Hantaan virus-induced hemorrhagic fever with renal syndrome. *Clin Immunol* 2006; 119(3): 245-51.

75. Li Y., Quan C., Xing W., et al. Rapid humoral immune responses are required for recovery from haemorrhagic fever with renal syndrome patients. *Emerg Microbes Infect* 2020; 9(1): 2303-14.

76. Tang K., Hou Y., Cheng L., et al. Increased blood CD226(-) inflammatory monocytes with low antigen presenting potential correlate positively with severity of hemorrhagic fever with renal syndrome. *Ann Med* 2023; 55(2): 2247000.

77. Zhang Y., Liu B., Ma Y., et al. Hantaan virus infection induces CXCL10 expression through TLR3, RIG-I, and MDA-5 pathways correlated with the disease severity. *Mediators Inflamm* 2014; 2014: 697837.

78. Zhang J., Tang K., Zhang Y., et al. The Presence of Circulating Nucleated Red Blood Cells Is Associated With Disease Severity in Patients of Hemorrhagic Fever With Renal Syndrome. *Front Med (Lausanne)* 2021; 8: 665410.

79. Liu B., Ma Y., Yi J., et al. Elevated plasma soluble Sema4D/CD100 levels are associated with disease severity in patients of hemorrhagic fever with renal syndrome. *PLoS One* 2013; 8(9): e73958.

80. Zhang H., Wang Y., Ma Y., et al. Increased CD4(+)CD8(+) Double Positive T Cells during Hantaan Virus Infection. *Viruses* 2022; 14(10).

81. Wang J., Guo W., Du H., et al. Elevated soluble CD163 plasma levels are associated with disease severity in patients with hemorrhagic fever with renal syndrome. *PLoS One* 2014; 9(11): e112127.

82. Wang M., Wang J., Wang T., Li J., Hui L., Ha X. Thrombocytopenia as a predictor of severe acute kidney injury in patients with Hantaan virus infections. *PLoS One* 2013; 8(1): e53236.

83. Liu B., Ma Y., Zhang Y., et al. CD8low CD100- T Cells Identify a Novel CD8 T Cell Subset Associated with Viral Control during Human Hantaan Virus Infection. *J Virol* 2015; 89(23): 11834-44.

84. Li J., Liu Y. X., Zhao Z. T. Genotyping of hantaviruses occurring in Linyi, China, by nested RT-PCR combined with single-strand conformation polymorphism analysis. *Acta Virol* 2009; 53(2): 121-4.

85. Ma Y., Yuan B., Yi J., et al. The genetic polymorphisms of HLA are strongly correlated with the disease severity after Hantaan virus infection in the Chinese Han population. *Clin Dev Immunol* 2012; 2012: 308237.

86. Zhang Z. H. Antibody levels and serotyping of patients with hemorrhagic fever of renal syndrome in Changchun region. *Chinese Journal of Public Health Engineering* 2006; (06): 370+2.

87. Tang K., Zhang C., Zhang Y., et al. Elevated plasma interleukin 34 levels correlate with disease severity-reflecting parameters of patients with haemorrhagic fever with renal syndrome. *Infect Dis (Lond)* 2019; 51(11-12): 847-53.

88. Tang K., Zhang C., Zhang Y., et al. Elevated Plasma Soluble CD14 Levels Correlate with the Monocyte Response Status During Hantaan Virus Infection in Humans. *Viral Immunol* 2015; 28(8): 442-7.

89. Wang M. L., Lai J. H., Zhu Y., et al. Genetic susceptibility to haemorrhagic fever with renal syndrome caused by Hantaan virus in Chinese Han population. *Int J Immunogenet* 2009; 36(4): 227-9.

90. Xie M., Dong Y., Zhou Y., Ren H., Ji Y., Lv S. Levels of HTNV-specific CD8+ T lymphocytes in PBMC from the patients with hemorrhagic fever with renal syndrome. *Intern Emerg Med* 2013; 8(6): 503-8.

91. Zhang Y., Ma Y., Zhang C., et al. Soluble Scavenger Receptor CD163 Is Associated with Severe Acute Kidney Injury in Patients with Hantaan Virus Infection. *Viral Immunol* 2015; 28(4): 241-6.

92. Li X., Du N., Xu G., et al. Expression of CD206 and CD163 on intermediate CD14(++)CD16(+) monocytes are increased in hemorrhagic fever with renal syndrome and are correlated with disease severity. *Virus Res* 2018; 253: 92-102.

93. Li P., Zhang C., Wang M., et al. Elevation of Myeloperoxidase Correlates with Disease Severity in Patients with Hantaan Virus Infection. *Viral Immunol* 2022; 35(6): 418-24.

94. Zhang C., Tang K., Zhang Y., et al. Elevated Plasma Fractalkine Level Is Associated with the Severity of Hemorrhagic Fever with Renal Syndrome in Humans. *Viral Immunol* 2021; 34(7): 491-9.

95. Wu P., Jiang W. J., Zhou J. Z., et al. Etiological detection of serum samples from patients with hemorrhagic fever with renal syndrome (HFRS) and rodent lung samples from high incidence areas of Guizhou Province in 2013 and 2014. *Chinese Journal of Hygienic Insecticides & Equipments* 2022; 28(05): 443-6.

96. Ma Y. X. Complete genome sequence and viral characteristics analysis of the Hantaan strains isolated from Shaanxi Province [Degree of master]; 2016.

97. Chai N. M., Tan Q. L., Shu J. W., et al. Host animal investigation and etiological analysis of Hantavirus in some areas of Zhoushan Islands, Zhejiang province, China, 2021. *Chinese Journal of Vector Biology and Control* 2023; 34(03): 326-30.

98. Luo X. H., Huang S. J., Chen L. F., Li Y. D., Gao H. An investigation on the infection status and genotype of Hantavirus carried by rodents in Yuyao City of Zhejiang Provinc. *Preventive Medicine* 2016; 28(08): 789-91+95.

99. Zhou P. C., Y. C., Yang X. D. Investigation of the source of hemorrhagic fever with renal syndrome in Youxi County and virus isolation. *Strait Journal of Preventive Medicine* 2011; 17(05): 35-6.

100. Liang C. Y., Xiong Y., Yao W. D., Qiu J. C., Di B. Identification of a strain of hemorrhagic fever with renal syndrome virus. *Chinese Journal of Vector Biology and Control* 2004; (03): 229-30.

101. Wang W., Dong J., Jia Y. H., et al. Investigation of rodents and rodent⁃borne pathogen in Jixian of Tianjin, China. *Chinese Journal of Vector Biology and Control* 2013; 24(03): 257-9.

102. Chen C. P., You C. H. Epidemiologic investigation and analysis ofhemorrhagic fever with renal syndrome in Songxi County, China. *South China Journal of Preventive Medicine* 2006; (03): 77-8.

103. Wang W. L., Xu Y. C., Yang D., et al. Analysis on the Situation of Hantavirus Carried by Rodents at Shuangmufeng Port. *China Port Science and Technology* 2022; 4(10): 20-4.

104. Li G. Y., Chen Y. P., Pan L., et al. Sero-epidemiological survey of hemorrhagic fever with renal sydrome in natural epidemic areas of Yan'an district od Shaanxi province. *Chinese Journal of Zoonoses* 2004; (06): 495-9.

105. Ke X. M., Wang X. Q., Guo Z. N., Chen H. F., Wu S. H., He W. Q. Detection of Hantaviruses in rat-like animals in Haicang region, Xiamen city. *Journal of Tropical Medicine* 2022; 22(12): 1738-40.

106. Qu Y. G., Yang G. Q., Zou Y., Yan G. G., Chen H. X., Zhang Y. Z. Isolation and characterization of Hantavirus carried by rodents in Huludao, Liaoning Province. *Chinese Journal of Epidemiology* 2006; (06): 513-7.

107. Wang Y. L., Yang Y. L. Analysis of monitoring results of hemorrhagic fever with renal syndrome in Jinjiang City from 2011 to 2012. *Strait Journal of Preventive Medicine* 2013; 19(06): 41-2.

108. Liu S. W., Xu G., Shi Y., et al. Hantavirus isolation and the complete S, M segments genetic analysis of the isolates in Jiangxi Province, China. *Chinese Journal of Zoonoses* 2015; 31(12): 1157-61+99.

109. Shen B., Wu D. L., Piao D. F., et al. Isolation and identification of three strains of seoul virus in Hunchun City of Jilin Province. *Chinese Journal of Public Health Engineering* 2008; (03): 167-9.

110. Liu B. Y., Ni X. G., Ran C. D., et al. Genetic analysis of M segment in hantaviruses carried by wild rodents from the Huanggang area, Hubei province. *E-Journal of Translational Medicine* 2017; 4(04): 48-51.

111. Wang Y., Wei Y. M., Han X., et al. Host rodent and gene characteristics of Hantavirus in main endemic areas of hemorrhagic fever with renal syndrome in Hebei province. *Chinese Journal of Vector Biology and Control* 2017; 28(06): 553-6.

112. Weng J. Q., Xie R. H., Yao P. P., et al. Full length nucleotide sequence analysis of L segments of Hantavirus ZT10 strain gene Compilation of papers from the 2007 Zhejiang Provincial Academic Annual Conference on Medical Virology, Medical Microbiology and Immunology; 2007; Zhejiang Zhoushan, China; 2007. p. 4.

113. Lin X. D., Guo W. P., Wang W., et al. Prevalence of Hantavirus infections in humans and animals in Wenzhou city. *Chinese Journal of Vector Biology and Control* 2010; 21(03): 235-7.

114. He S., Chen Y., Wang L. L., et al. Surveillance and analysis on HFRS epidemic situation and the host animals in Fujian Province. *Strait Journal of Preventive Medicine* 2011; 17(02): 8-10.

115. Pang Z. Q., Pang R. X., Lu S. H., Yemin Q., Zhenwang Z. Monitoring and analysis on the host animals of hemorrhagic fever with renal sydrome in Cangzhou City. *Journal of Medical Pest Control* 2010; 26(06): 531+3.

116. Jiang J. F., Wu X. M., Wang R. M., et al. Epidemiologic Investigation on Hantaviruses Carried by Rodents in Urban District of Beijing. *Chinese Journal of Vector Biology and Control* 2006; (02): 93-7.

117. Tang F., Li S. L., Wang A. G., et al. Study on ecological epidemiology of Hantavirus reservoirs in Beijing city. *Chinese Journal of Public Health* 2006; (04): 480-1.

118. Tao X., Peng X., Jia C. H., Yongsong L., Mingkun S., Lixin Z. Surveillance on host animals of hemorrhagic fever with renal syndrome in Qinhuangdao City from 2005-2013. *Occupation and Health* 2015; 31(21): 2986-8.

119. Gao Y. F., Song G. R., Tan Z., Lu J., Xiaolan C., Fenglin S. Surveillance and detection of rodents and hantavirus in Liaoning port from 2008 to 2015. *Chinese Journal of Frontier Health and Quarantine* 2017; 40(02): 100-3.

120. Song F. L., Wu G., Xue F., et al. Study on Pathogens Carried by Vectors at Frontier Port. *Chinese Journal of Frontier Health and Quarantine* 2009; 32(05): 368-74.

121. Bi F. Y., Tan Y., Mo J. J., Zhenguo X., Minmei C. Genotypes of hantanvirus in north areas of Guangxi. *Applied Preventive Medicine* 2016; 22(05): 383-5+97.

122. Li Y., Shi L. L., Liu J. N., Jihuan Y., Weizhong N., Shuping L. S gene sequence analysis of Hantavirus carried by the Rattus norvegicus from Qinhuangdao port. *Chinese Journal of Vector Biology and Control* 2017; 28(05): 487-9.

123. Xiong H. P., Li M. H., Zhu Y., et al. Molecular epidemiology of hantavirus carried by rodent hosts in Wuhan，Hubei Province. *China Tropical Medicine* 2010; 10(06): 658-60.

124. Zhang X., Ma M. X., Zhang Z., et al. Molecular epidemiology of hantavirus isolated from rodent hosts in Jinzhou city. *Chinese Journal of Public Health* 2015; 31(01): 39-41.

125. Hu T., Fan Q., Hu X., et al. Molecular and serological evidence for Seoul virus in rats (Rattus norvegicus) in Zhangmu, Tibet, China. *Arch Virol* 2015; 160(5): 1353-7.

126. Liu D. Y., Liu J., Liu B. Y., et al. Phylogenetic analysis based on mitochondrial DNA sequences of wild rats, and the relationship with Seoul virus infection in Hubei, China. *Virol Sin* 2017; 32(3): 235-44.

127. Guo G., Sheng J., Wu X., et al. Seoul virus in the Brown Rat ( Rattus norvegicus ) from Ürümqi, Xinjiang, Northwest of China. *J Wildl Dis* 2016; 52(3): 705-8.

128. Wei Y. M., Han Z. Y., Zhang Y. B., et al. The isolation, recovery and identification of Hantavirus in Hebei province. *Chinese Journal of Vector Biology and Control* 2016; 27(05): 447-9+58.

129. Gao Y. X., Li X. B., Fang S. F., et al. Investigation of Yersinia pestis and Hantavirus carried by small mammal hosts at Guangdong entry⁃exit ports. *Chinese Journal of Vector Biology and Control* 2016; 27(02): 137-40.

130. Chen L. W., Yue Q. Y., Qiu D. Y., Hanhan Y., Dexing L., Qiuxing H. Investigation on the pathogens carried by rodents at Shenwan port in Zhongshan city. *Chinese Journal of Frontier Health and Quarantine* 2019; 42(01): 33-6.

131. He J. Q., Ke Y. Z., Tang Z. Z., Yu Y. S. Investigation on rodents at Xiaocuo port. *Chinese Journal of Frontier Health and Quarantine* 2013; 36(02): 96-8+103.

132. Li B. X., Ma S. T., An J. L., et al. Investigation on natural infection of rodent hantavirus in Ji'an port area. *Port Health Control* 2012; 17(03): 27-9.

133. Zhao S., Chen Z. Q., Zhan Z. H., Ye T. T., Wang X. Y. Investigation on Hantaviruses carried by rodents in Nansha ports. *Chinese Journal of Vector Biology and Control* 2016; 27(06): 591-3.

134. Hou Y., Li D. P., Liu Y. P., et al. Epidemiologic investigation and analysis on Hantavirus carried by rodents at Heilongjiang frontier ports between Sino-Russian border in 2009-2010. *Chinese Journal of Frontier Health and Quarantine* 2012; 35(03): 186-9.

135. Yin X. P., Song F. L., Zhao S. S., et al. Hantavirus detected from small mammals in the China⁃Kazakhstan border. *Chinese Journal of Vector Biology and Control* 2018; 29(01): 38-41.

136. Geng Y. Z., Tian J., Liu Y., et al. Genetic subtypes and distribution of Seoul hanta virus in Liaoning province. *Chinese Journal of Public Health* 2012; 28(12): 1594-6.

137. Bao H. M., Wei Y. H., Yuan L. H., Shouyi C., Zhicong Y., Jiahai L. The detection and genotyping of hantavirus in small mammals in Guangzhou. *Journal of Tropical Medicine* 2018; 18(05): 565-8.

138. Zhao C., Zhao Y., Li Y., et al. Genotype Analysis of HFRS Pathogen in Jilin Area. *Journal of Microbiology* 2011; 31(05): 101-4.

139. Sun B. X., Sun Y., Shen B., Wu J. Genotype analysis of Hantavirus carried by the rodents in Changchun city. *Chinese Journal of Vector Biology and Control* 2015; 26(06): 600-4.

140. Fan S. T., Gao X. L., Li Y. G., et al. Genetics and evolution of viruses carried by animal hosts of Hantaviruses in Jilin Province，China. *Chinese Journal of Biologicals* 2014; 27(04): 467-71+75.

141. Su Q., Chen Y., Li M., et al. Genetic Characterization and Molecular Evolution of Urban Seoul Virus in Southern China. *Viruses* 2019; 11(12).

142. Wang Y., Cao S., Cheng C., Ju W., Hua Y. Genetic characterization of a hantavirus isolated from Heilongjiang province, China. *Can J Vet Res* 2019; 83(1): 75-7.

143. Wang Q. W., Tao L., Lu S. Y., et al. Genetic and hosts characterization of hantaviruses in port areas in Hainan Province, P. R. China. *PLoS One* 2022; 17(3): e0264859.

144. Yan Q. G., Xing Y. Z., Li B. B., et al. Genetic characteristics of Seoul orthohantavirus in rodents in Huai’an，Jiangsu province. *Chinese Journal of Frontier Health and Quarantine* 2019; 42(04): 254-8+83.

145. Du Y. H., Li Y., Ma H., Haifeng W., Bianli X., Xueyong H. Etiological analysis of hantavirus in rats in Henan Province from 2014 to 2016. *Tianjin Medical Journal* 2017; 第45卷(第6期): 648-51.

146. Han X., Han Z. Y., Wei Y. M., et al. Epidemiology of hemorrhagic fever with renal syndrome in Hebei province in 2012. *China Tropical Medicine* 2014; 14(1): 57-9.

147. Chen Y., Lin D. H., Chen L., et al. Epidemiological surveillance of hemorrhagic fever with renal syndrome and associated reservoir hosts during 2012 in Fujian province, China. *Chinese Journal of Vector Biology and Control* 2014; (2): 177-9.

148. Hu T. S., Hu Q. L., Li S. X., et al. Epidemiological features of hemorrhagic fever with renal syndrome and the host animals in Chuxiong, Yunnan province, China, 2015-2018. *Chinese Journal of Vector Biology and Control* 2020; 31(02): 152-7+63.

149. Liu S. W., Xu G., Gong T., et al. The detection and genotyping of hantavirus in rats in Nanchang City. *Modern Preventive Medicine* 2016; 43(06): 1085-8+96.

150. Guo T. Epidemic status of hemorrhagic fever with renal syndrome and investigation of host animals in some areas in Yunnan Province from 2014 to 2015 [Degree of master]; 2016.

151. Wang B., Cai C. L., Li B., et al. Detection and characterization of three zoonotic viruses in wild rodents and shrews from Shenzhen city, China. *Virol Sin* 2017; 32(4): 290-7.

152. He W., Fu J., Wen Y., Cheng M., Mo Y., Chen Q. Detection and Genetic Characterization of Seoul Virus in Liver Tissue Samples From Rattus norvegicus and Rattus tanezumi in Urban Areas of Southern China. *Front Vet Sci* 2021; 8: 748232.

153. Cao Y. C., Gao R., Wang L. X., Li M. Detection and analysis of hantavirus antigen in Taonan City, Jilin Province in 2013. *Chinese Journal of Control of Endemic Diseases* 2015; 30(04): 272.

154. Chai W. L., Wang Y. P., Zhang J. M., et al. Analysis on pathogens monitoring results of rodent at Fujian port from 2010 to 2012. *Chinese Journal of Frontier Health and Quarantine* 2013; 36(04): 223-7.

155. Han X., Han Z. Y., Wei Y. M., Yanbo Z., Shunxiang Q., Qi L. Analysis of the surveillance data on host animals in national monitoring sites of hemorrhagic fever with renal syndrome in Hebei province in 2011. Chinese Journal of Health Laboratory Technology 2013; 23(15): 3109-11+13.

156. He X., Qi C. G., Wang C., Jiang Y. Z. Analysis of rat monitoring results at Lianyungang port from 2014 to 2015. Port Health Control 2016; 21(05): 41-4.

157. Chen L. F., Luo X. H., Chen J. Analysis of monitoring results of hemorrhagic fever with renal syndrome in Yuyao City from 1996 to 2015. China Preventive Medicine Journal 2016; 28(07): 697-9.

158. Luo Y. N. Analysis on the surveillance results of hemorrhagic fever with renal syndrome in Cixi city from 1996 to 2011. Chinese Journal of Vector Biology and Control 2012; 23(03): 265-7.

159. Xu Q., Wang Y. P., Gao B., Jianming Z., Yanping Z., Hengzhong C. Analysis of monitoring results of hemorrhagic fever with rat renal syndrome at Fujian port in 2010. Port Health Control 2011; 16(06): 43-5.

160. Liu D. P., Wan Q., Qu R. W., et al. Analysis on genetic characteristic hantavirus carrying by rats, Dalian city, 2012. Preventive Medicine Tribune 2013; 19(12): 893-5+903.

161. Shang C., Yang L. F., Du S. S., et al. Analysis of Hantavirus Genotypes Carried by Rodents in Some Areas of Yunnan Province, 2018-2019. Chinese Journal of Virology 2022; 38(01): 149-55.

162. Xuan R. L., Huang J. F., Huang C. Q., Wenqing C., Xiaokang S., Yuanling D. Prevalence of epidemic hemorrhagic fever and monitoring of pathogens and vectors in Qingyuan City, 2002-2009. South China Journal of Preventive Medicine 2012; 38(01): 32-5.

163. Li Y. L. Epidemic development analyse of hemorrhagic fever with renal syndrome from 1992 to 2006 in Taian city. Journal of Preventive Medicine Information 2008; (06): 476-8.

164. Wei S. H., Wang J., Chu H. N., Jing S., Guoliang Z., Qiang Z. Epidemiological investigation on hemorrhagic fever with renal syndrome in Chengde city from 1984 to 2008. Chinese Journal of Vector Biology and Control 2010; 21(06): 606-8.

165. Meng X. Z., Chen Y. P., Li C., et al. Using recombinant antigens of Hantavirus to study the kinetics of serum IgA‚IgG‚IgM antibodies in the acute-phase of hemorrhagic fever renal syndrome. Chinese Journal of Experimental and Clinical Virology 2003; (03): 55-8.

166. Fu M. Molecular epidemiology of Hemorrhagic Fever with Renal Syndrome in Xiangyun Country, Yunnan Province [Degree of master]: Kunming Medical University; 2021.

167. Gao N., Ma C., Yang P. F., Li M. H., Zhang Y. Z. Analysis of infectious status of hemorrhagic fever with renal syndrome in Bayanzhuoer City. China Tropical Medicine 2008; (11): 1891-3+9.
